# Galectin-9 binding to HLA-DR in dendritic cells controls immune synapse formation and T cell proliferation

**DOI:** 10.1073/pnas.2501381122

**Published:** 2025-12-08

**Authors:** Andrea Rodgers-Furones, Thijs Brands, Guusje van Gameren, Mirane Florencio-Zabaleta, Mayukha Bathini, Sandra Delgado, Zacharias Wijfjes, Kristina Fedorova, René Classens, Lona Kroese, Martijn Verdoes, Guido van Mierlo, Jesús Jiménez-Barbero, Rik G. H. Lindeboom, Ana Ardá, Annemiek B. van Spriel, Laia Querol Cano

**Affiliations:** ^a^Department of Medical BioSciences, Radboudumc, Nijmegen 6525GA, Netherlands; ^b^Centro de Investigación Cooperativa en Biociencias, Basque Research Technology Alliance, Bizkaia Technology Park, Derio 48160, Spain; ^c^Netherlands Cancer Institute, Amsterdam 1066CX, Netherlands; ^d^Mouse Clinic for Cancer and Aging research, Transgenic Facility, Netherlands Cancer Institute, Amsterdam 1066CX, Netherlands; ^e^Ikerbasque, Basque Foundation for Science, Bilbao 48009, Spain; ^f^Centro de Investigación Biomedica en Red de Enfermedades Respiratorias, Madrid 28029, Spain; ^g^Department Organic and Inorganic Chemistry, Universidad del País Vasco/Euskal Herriko Unibertsitatea, Leioa 48940, Spain

**Keywords:** immune synapse, galectin, dendritic cell, HLA-DR

## Abstract

To initiate T cell–mediated immunity, dendritic cells (DCs) present antigens to T cells through the establishment of an immune synapse (IS). While events behind IS formation in T cells are well understood, the organization of IS components at the DC membrane remains ill-defined. Galectins modulate immune responses via glycan-(in)dependent interactions but the molecular mechanisms underlying their function in DC–mediated T cell activation, are poorly described. Here, we demonstrate that intracellular galectin-9 (gal9) in DCs is required for CD4^+^ T cell activation through its interaction with the HLA-DR alpha- and beta- cytoplasmic domains, as supported by coimmunoprecipitation, mass spectrometry, and NMR analyses. Live-cell imaging revealed gal9-depleted DCs fail to establish stable ISs with T cells, reducing T cell activation and proliferation. Notably, HLA-DR membrane lateral mobility and recruitment to the IS was diminished in DCs lacking gal9. Conditional gal9 knockout in DCs led to enhanced tumor growth in vivo, underscoring a role for gal9 in T cell–dependent antitumor immunity. Collectively, this study reports gal9-mediated HLA-DR organization at the DC synapse, uncovering a mechanism through which intracellular galectins coordinate immune receptor positioning to enhance CD4^+^ T cell activation.

## Significance

This research uncovers an important role for galectin-9 (gal9) in dendritic cells (DCs) during the engagement and subsequent activation of CD4^+^ T cells, which is indispensable to initiate adaptive immunity. Gal9 expressed by DCs aids in the establishment of durable interactions with T cells by properly displaying activating signals (MHC-II molecules) at the contact site between both cells (immune synapses, IS). Without gal9, DCs fail to efficiently stimulate CD4^+^ T cells, diminishing subsequent immune responses, with consequences for antitumor immunity. This study reveals an immune-regulating mechanism mediated by galectins, which can aid in understanding disbalanced immune responses, such as cancer.

Dendritic cells (DCs) are paramount to initiate cellular adaptive immunity due to their ability to recognize, digest, and present antigens or tumor cells as peptides to CD8^+^ or CD4^+^ T cells *via* the Major Histocompatibility complex-I or -II (MHC), respectively (1, 2).

Antigen-specific interactions between DCs and T cells lead to the formation of the immune synapse (IS), a highly ordered structure indispensable for T cell activation and function (3). Assembly of an IS requires the recruitment of membrane receptors and signaling components to spatiotemporally organized domains at the interface between both cells (4). Initial IS formation is dependent on the clustering of MHC-I or -II and of the T cell receptor (TCR) (5). This enables the recruitment of costimulatory molecules, integrins, and other surface receptors that facilitate downstream signaling and dictate the strength and type of T cell immune responses (4, 6). The canonical organization of the IS consists of a TCR-MHC-rich central supramolecular activation cluster (c-SMAC), surrounded by a peripheral SMAC (p-SMAC) containing adhesion molecules (LFA-1/ICAM-1) that provide a mechanical scaffold for the IS and a distal SMAC (d-SMAC) that contains immunoinhibitory receptors such as the tyrosine phosphatase CD45 (7). In contrast to the unifocal T cell–B cell synapse, the structure of the DC-T cell synapse appears to be multifocal, containing numerous segregated microdomains in which IS components are distributed at different points of the membrane, enabling multiple cell–cell interaction sites (5, 8, 9). The first phase of DC-T cell interactions consists of transient intermittent contacts that last approximately 10 to 12 min followed by a second phase, marked by the formation of stable DC clusters interacting with multiple T cells simultaneously, which can extend for about 2 to 3 h (3). Upon DC-T cell disengagement, a third phase follows where T cells regain their motility and proliferate (3). Importantly, DC-T cell interactions rely on the reorientation of the microtubule organizing center and on cytoskeletal remodeling in DCs to drive the recruitment of IS components to specific areas within the contact zone and to promote DC polarization toward the interacting T cells (10–12). Nonetheless, while the molecular mechanisms governing IS membrane organization on the T cell side are well understood, the organization of the IS on the DC side remains ill-defined.

Galectins are a family of soluble β-galactoside binding proteins that share at least one conserved carbohydrate recognition domain (CRD). Galectins modulate receptor mobility, clustering, or stability through specific carbohydrate-dependent interactions on the cell surface and also exert intracellular functions through both glycan-independent and glycan-dependent interactions (13, 14). In DCs galectins can play diverse roles. For example, galectin-1 (gal1) endows DCs with tolerogenic properties, modulating their ability to induce T cell tolerance (15). Galectin-3 (gal3) affects DC migration, cytokine production, and the balance of Th17 responses (16). Galectin-9 (gal9) promotes cytokine secretion, phagocytosis, maturation and enhances Th1 responses (17–19).

At the IS, galectins expressed by T cells contribute to the formation of structured surface domains, also referred to as lattices, such as the N-glycosylated TCR lattice in T cells, which prevents unintended activation in resting cells (20). For instance, extracellular gal3 inhibits T cell activation by restricting the lateral movement of the TCR within the IS and dissociating CD8 from the TCR (21). Additionally, galectin-3 influences synapse dynamics by sequestering LFA-1 in membrane structures, thereby affecting LFA-1 recruitment and activation (22, 23). Illustrating the pleiotropic functions of galectins in T cells, intracellular gal9 enhances T cell activation by supporting TCR–CD3 complex formation and signaling pathways (24, 25) whereas gal3 triggers TCR internalization effectively inhibiting T cell activation (23). Despite these well-characterized functions on T cells, the role of galectins at the DC side of the IS remains unexplored.

Here, we demonstrate that DCs require gal9 to interact with CD4^+^ T cells. We identified an intracellular interaction between gal9 and MHC-II (HLA-DR) crucial for its recruitment to the contact zone between DC and T cells and subsequent T cell activation and proliferation. Our work reveals an important function for intracellular gal9 in regulating DC-IS formation, important for T cell activation in vivo.

## Results

### Gal9 Is Required for CD4^+^ T cell Activation.

To determine in which processes gal9 is involved in human DCs in an unbiased manner, we examined transcriptional changes following gal9 knockdown. Monocyte-derived DCs were transfected with either nontargeting siRNA or *Lgals9* siRNA (referred to as WT and KD gal9 DCs, respectively) (*SI Appendix*, Fig. S1*A*). Gal9 surface expression was nearly completely abolished without affecting DC maturation (*SI Appendix*, Fig. S1*B* and *C*). RNA-seq analysis of WT and KD gal9 DCs from three independent donors revealed broad transcriptional changes, with gal9 among the most downregulated genes (*SI Appendix*, Fig. S2*A*). Gene set enrichment was performed using average fold change across replicates as input to account for interdonor variation (*SI Appendix*, Fig. S2 *B* and *D*). Analysis revealed increased expression of genes involved in antigen processing via MHC-II, and reduction of gene expression programs involved in CD4^+^ T cell proliferation and activation upon gal9 depletion (*SI Appendix*, Fig. S2*C*). We then investigated the functional consequences of gal9 depletion in DC-mediated T cell activation using a mixed lymphocyte reaction (MLR) in which WT or KD gal9 DCs were cocultured with allogeneic T cells. Depletion of gal9 diminished T cell activation as seen by the lower expression of the activation marker CD25 upon coculture with KD gal9 DCs (*SI Appendix*, Fig. S3 *A* and S3 *B*). Concomitantly, T cells also exhibited lower IFN-γ secretion (*SI Appendix*, Fig. S3*C*) and proliferation capacity (*SI Appendix*, Fig. S3 *D* and *E*) when compared to T cells activated by WT DCs. Since gal9 in DCs is located both in the cytosol and extracellularly (membrane-bound) (19), we investigated the involvement of both fractions in driving T cell proliferation by treating KD gal9 DCs with exogenous recombinant gal9 (rgal9) prior to subjecting them to MLR assays. In addition, WT DCs were treated with a specific gal9 blocking antibody (17). Exogenous rgal9 restored surface-bound gal9 levels (*SI Appendix*, Fig. S3*F*) but did not rescue the impairment in T cell expansion. Concomitantly, blocking extracellular gal9 in WT DCs did not detrimentally alter T cell proliferation (*SI Appendix*, Fig. S3 *G* and *H*), suggesting the intracellular fraction of gal9 could be responsible for the diminished T cell induction.

**Fig. 1.**
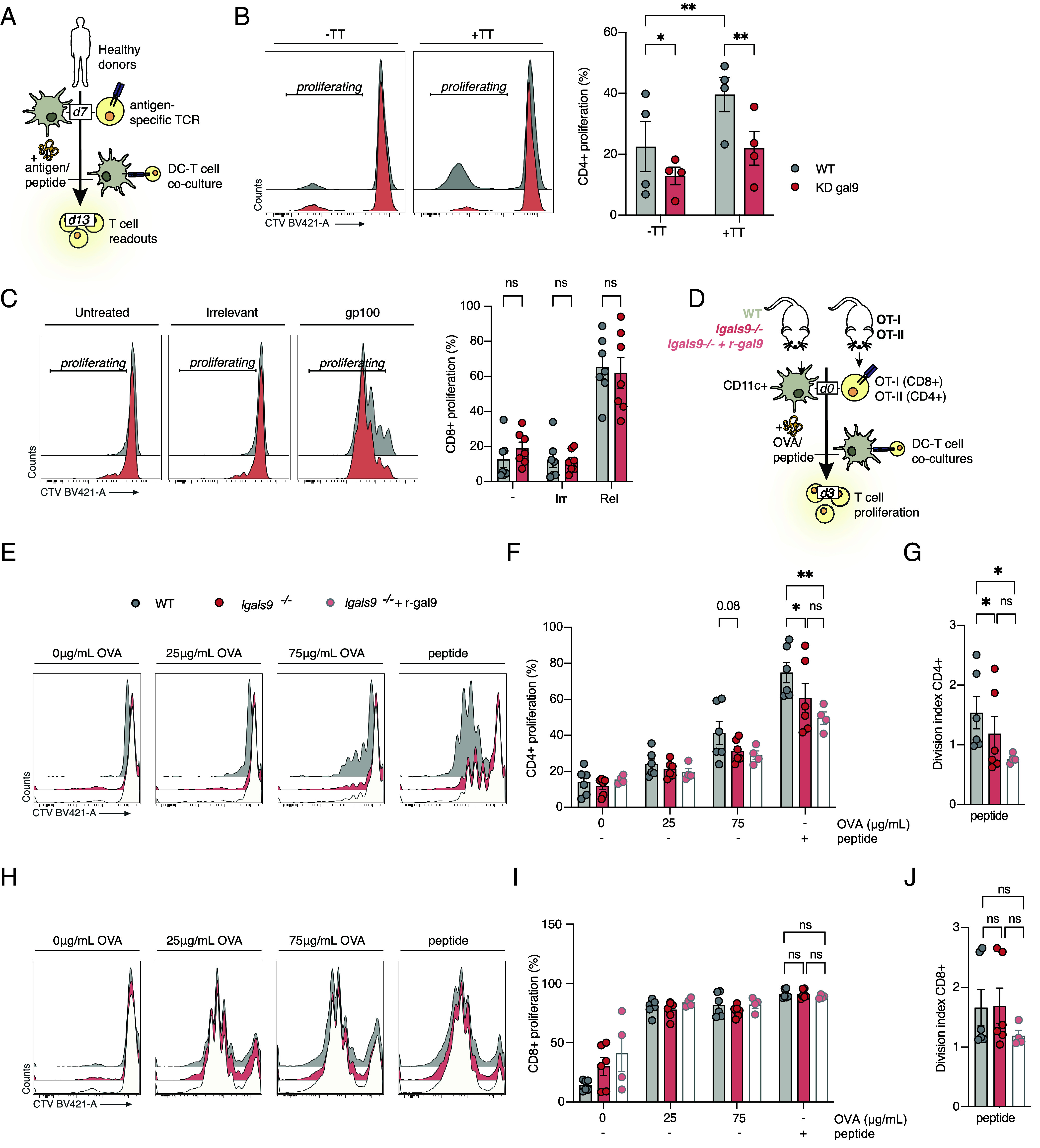
Gal9-deficient DCs are impaired in their ability to induce antigen-specific CD4^+^ T cell proliferation. (*A*) Schematic of human autologous T cell activation assay using WT or KD gal9 DCs and antigen-specific CD4^+^ [from vaccinated donors with Tetanus toxoid (TT)] or CD8^+^ (transfected with an mRNA encoding for gp100-TCR) T cells. (*B*) CD4^+^ T cell proliferation in response to TT or PBS (−TT) presented by WT (gray) or KD gal9 DCs (red). Representative flow cytometry histogram (*Left*) and quantification (n = 4, *Right*). (*C*) CD8^+^ T cell proliferation in response to PBS (−), irrelevant peptide (NY-ESO, Irr), or relevant peptide (gp100, Rel)-treated WT (gray) or KD gal9 DCs (red). Representative flow cytometry histogram (*Left*) and quantification (n = 7, *Right*). (*D*) Schematic of murine DC-T cell coculture assay using OVA-treated-WT, gal9 KO (*lgals9*^−/−^), or gal9-rescued KO cells (*lgals9*^−/−^ + rgal9) with OT-I or OT-II T cells. (*E* and *F*) OT-II CD4^+^ T cell proliferation in response to OVA protein or peptide presented by WT, *lgals9*^−/−^, or *lgals9*^−/−^ + rgal9 DCs; representative histograms (*E*) and quantification (n = 4 to 6 mice, *F*). (*G*) Division index of OT-II CD4^+^ T cells shown in (*F*). (*H* and *I*) OT-I CD8^+^ T cell proliferation in response to OVA protein or peptide presented by WT, *lgals9*^−/−^, or *lgals9*^−/−^ + r-gal9 DCs; representative histograms (*H*) and quantification (n = 4 to 6 mice, *I*). (*J*) Division index of OT-I CD8^+^ T cells shown in (*I*). Data shown as mean ± SEM. Each dot represents an independent donor (human) or mouse. Statistical significance assessed by two-way ANOVA with Šídák’s multiple comparisons test. ns *P* > 0.05, *P < 0.05, **P < 0.005.

We validated gal9 involvement in T cell activation using autologous antigen-driven models as they pose a more physiological setup (Fig. 1*A*). First, we studied CD4^+^ T cell activation using tetanus toxoid (TT) as antigen. TT-primed WT DCs induced TT-specific T cell proliferation, which was significantly inhibited by T cell coculture with KD gal9 DCs (Fig. 1*B*). Interestingly, nonspecific CD4^+^ T cell proliferation also decreased in the presence of KD gal9 DCs suggesting gal9-induced effects on T cell proliferation are not antigen specific (Fig. 1*B*). In contrast, autologous proliferation assays using the HLA-I-restricted peptide gp100 (280 to 288) revealed no effect of gal9 depletion in DC-mediated *^gp100+^*CD8^+^ T cell activation (Fig. 1*C* and *SI Appendix*, Fig. S4*A*). In agreement, perforin and granzyme B expression in gp100_TCR^+^ T cells as well as their TNFα and IL-2 production markedly increased upon activation with gp100-pulsed DCs regardless of gal9 expression (*SI Appendix*, Fig. S4*B*). Similarly, activation markers CD25, CD69, and CD137 were upregulated upon coculture but showed no difference between WT and KD gal9 DCs (*SI Appendix*, Fig. S4*C*). T cell cocultures with untreated DCs or with DCs pulsed with an irrelevant peptide induced no T cell proliferation or activation confirming the specificity and robustness of our data (Fig. 1*C* and *SI Appendix*, Fig. S4 *B* and *C*).

Next, we investigated whether gal9 function in T cell activation was conserved across species by using ovalbumin (OVA) on splenic DCs (CD11c^+^) isolated from WT and gal9^−/−^ (KO) mice. CD8^+^ and CD4^+^ T cells were harvested from TCR transgenic OT-I and OT-II mice respectively and stimulated in vitro with WT or gal9 KO DCs primed with either increasing concentrations of OVA or peptide (Fig. 1*D*). CD4^+^ T cell proliferation and the division index (the number of divisions each T cell underwent), was reduced in CD4^+^ T cells activated by gal9 KO DCs compared to WT DCs (Fig. 1 *E*–*G*), whereas neither CD8^+^ T cell proliferation nor their division index was affected by gal9 depletion in line with human DC data (Fig. 1 *H*–*J*). In addition, extracellular treatment of gal9 KO DCs with murine rgal9 did not alter CD4^+^ (Fig. 1 *F* and *G*) nor CD8^+^ (Fig. 1 *I* and *J*) T cell proliferation or cell division index.

Overall, these data demonstrate that gal9 in DCs is required for CD4^+^ T cell proliferation and activation.

### Gal9 Is Not Involved in Antigen Processing or Presentation.

Next, we examined whether gal9 could modulate antigen processing and presentation. Human WT or KD gal9 DCs were treated with increasing concentrations of self-quenching OVA (DQ-ova) (26), which emits fluorescence upon proteolytic degradation. No differences in cleaved DQ-ova fluorescence signal were observed between WT or KD gal9 DCs pulsed with increasing DQ-ova concentrations suggesting that gal9 is not involved in driving antigen processing (*SI Appendix*, Fig. S5 *A* and *B*). Pretreatment with Bafilomycin A, a lysosome inhibitor that blocks endosomal acidification, completely abrogated antigen processing in both WT and KD gal9 DCs, confirming the pH dependency of antigen degradation (*SI Appendix*, Fig. S5 *A* and *B*). To further corroborate that depleting gal9 does not compromise the proteolytic capacity of DCs, we examined cysteine cathepsin activity in WT and gal9-depleted murine and human DCs using the panreactive activity-based probe BMV109 (27). Incubation with the probe resulted in equal labeling intensities for all cysteine cathepsins detected in both WT and gal9-depleted cells (*SI Appendix*, Fig. S5 *C* and *E*). Similarly, treatment with MV151 (28), an activity-based probe targeting the immuno- and constitutive proteasome also revealed equal activity of labeled catalytic β and βi subunits (*SI Appendix*, Fig. S5 *D* and *F*) in both human and murine gal9-depleted DCs compared to their WT counterparts.

**Fig. 2.**
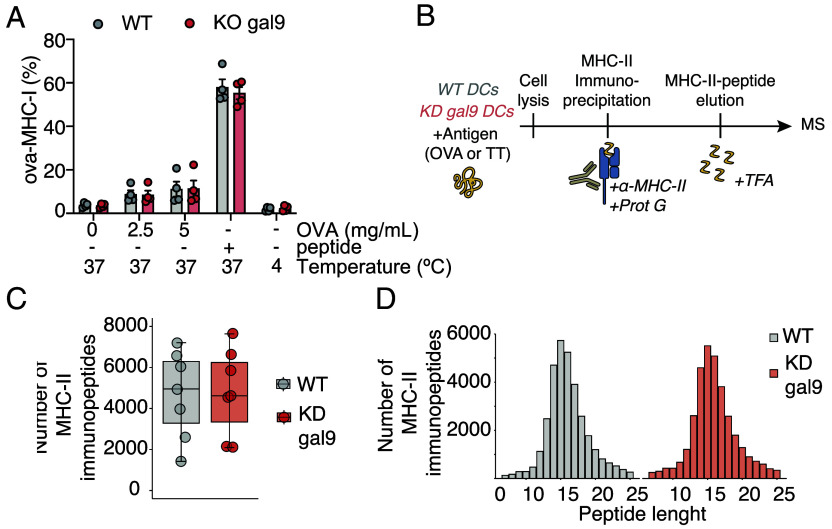
MHC class I and II–peptide complexes in WT DCs do not differ from gal9 KD DCs. (*A*) Fluorescence intensity quantification of SIINFEKL peptide bound to MHC-I detected in WT and gal9 KO murine DCs after culturing them with 0, 2.5, or 5 mg/mL OVA (or peptide as positive control) at 37 °C or 4 °C for 18 h (n = 4 mice). (*B*) Workflow for MHC-II-bound peptide isolation and analysis by mass spectrometry. Cell pellets from WT and gal9 KD DCs treated with OVA or TT antigens were subjected to mild lysis, followed by overnight incubation with protein G magnetic beads bound to anti-HLA-II antibodies. Peptides bound to MHC-II molecules were eluted from the bead–antibody–HLA–peptide complex using trifluoroacetic acid (TFA), desalted using StageTips, and analyzed by liquid chromatography–tandem mass spectrometry (LC-MS/MS, here: MS). (*C*) Total number of peptides identified in WT (gray) and KD gal9 (red) DCs. Each dot represents a different donor. (*D*) Length distribution (number of amino acid residues) of identified peptides in WT and KD gal9 DCs.

To assess whether gal9 modulates MHC-antigen presentation, we pulsed murine WT or gal9 KO DCs with increasing concentrations of OVA and determined MHC-I-peptide occupancy by flow cytometry. No differences were observed in antigen binding to MHC-I in WT or gal9 KO DCs (Fig. 2*A* and *SI Appendix*, Fig. S5*G*), confirming gal9 is not involved in this process. Next, we analyzed the MHC-II-bound antigen repertoire in WT and KO DCs, pulsed with TT or ovalbumin, by mass spectrometry-based MHC-II immunopeptidomics (Fig. 2*B*). Antigens identified revealed no differences in the amount or length distribution of peptides bound to MHC-II complexes upon gal9 loss (Fig. 2 *C* and *D* and *SI Appendix*, Fig. S5*H*), indicating that gal9 depletion does not impair MHC-II antigen processing and presentation capacity.

### Immune Synapse Formation Is Dependent on gal9.

**Fig. 3.**
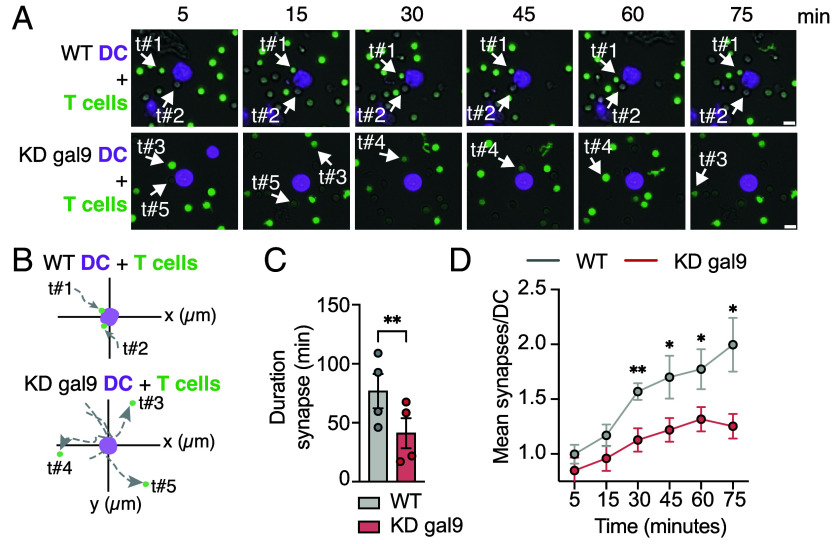
Gal9-deficient DCs fail to establish stable interactions with T cells. (*A*) Time-lapse images of superantigen treated-WT or KD gal9 DCs (magenta) cocultured with T cells (green) for the specified time points. White arrows illustrate tracked T cells (t#1-5) migrating toward DCs. (*B*) Migration tracks of individual T cells (t#1-5) in WT or KD gal9 DC–T cell cocultures, plotted across the x-y axis (μm). (*C*) Quantification of the synaptic contact duration (minutes) between T cells and WT (gray) or KD gal9 (red) DCs (n = 4 donors, 10 to 15 cells/donor). (*D*) Quantification of total DC–T cell contacts over time (n = 9 donors). (Scale bar, 10 μm.) Data are presented as mean values ± SEM. Statistical significance assessed by the unpaired *t* test analysis (*C*) or two-way ANOVA with Šídák’s multiple comparisons test (*D*). *P < 0.05, **P < 0.005.

We also studied the spatiotemporal dynamics of superantigen treated-(SEB)-WT or KD gal9 DC interactions with T cells using time lapse microscopy (*SI Appendix*, Fig. S6*A*). Gal9 depletion decreased the ability of DCs to establish stable cell–cell contacts with T cells (Fig. 3 *A* and *B* and Movies S1 and S2). Interactions between WT DCs and T cells were long-lasting (average duration >1 h) whereas KD gal9 DC–T cell interactions were transient, lasting approximately 40 min (Fig. 3*C*). Individual T cell tracks depict their enhanced motility in the vicinity of KD gal9 DCs compared to their suppressed locomotion upon contacting WT DCs (Fig. 3*B*). Gal9 depletion rendered DCs unable to establish simultaneous synaptic interactions with multiple T cells, forming so-called rosette structures necessary for proper T cell activation (8) (Fig. 3*D* and *SI Appendix*, Figs. S6 *B* and *C*). Overall these data show that gal9 in DCs is required for IS formation.

### Intracellular gal9 Binding to HLA-DR Directs Its Synaptic Localization and Membrane Mobility.

**Fig. 4.**
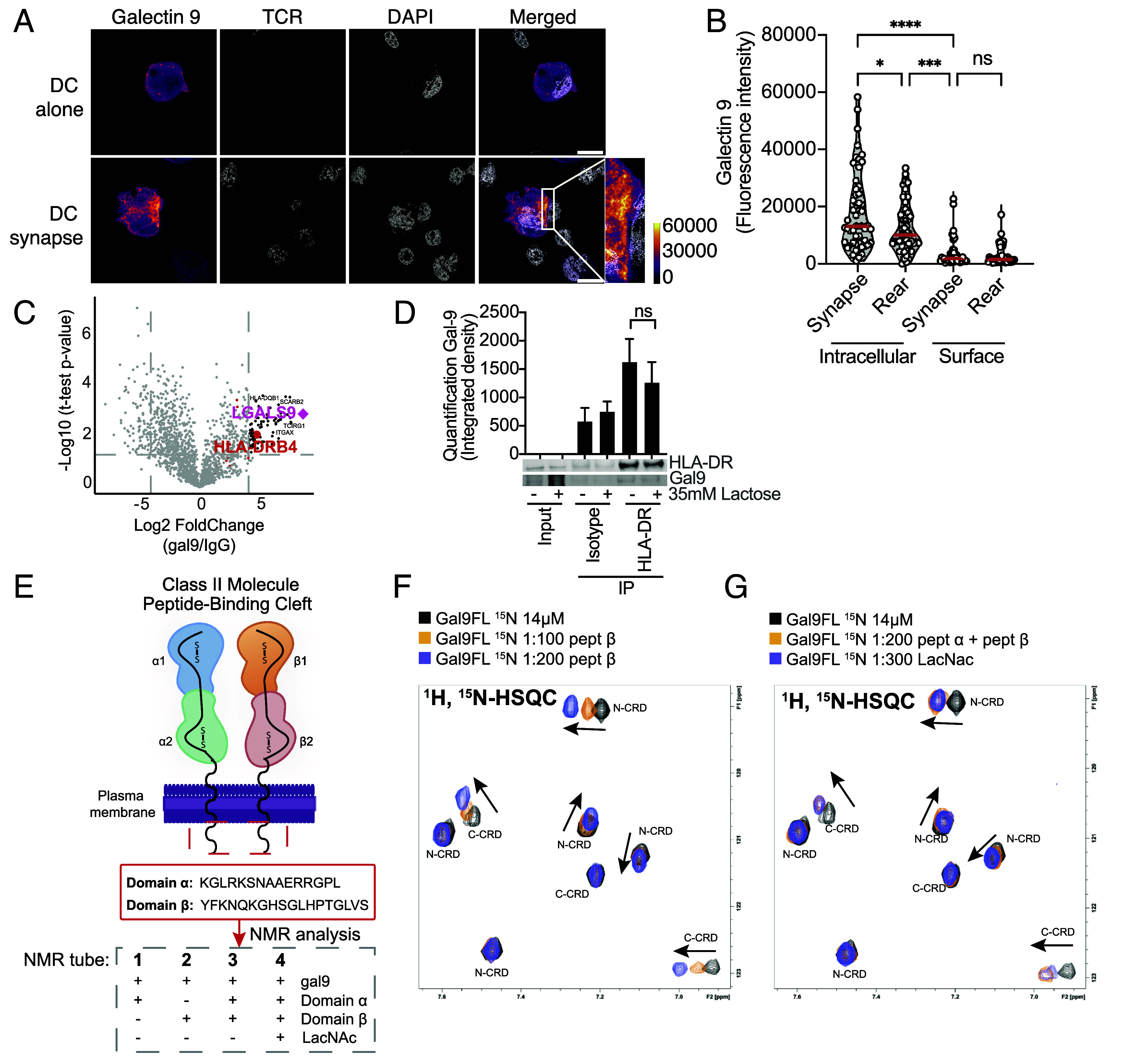
Gal9 localizes to the IS and interacts with the α and β intracellular domains of HLA-DR. (*A*) Representative immunofluorescence images of superantigen-treated DCs alone (*Top*) or cocultured with T cells (DC synapse, *Bottom*), permeabilized and stained against gal9, TCR, and DAPI. Merged and zoomed-in image highlights the localization of gal9 at the DC–T cell interface. Fluorescence intensity depicted as colorized pixels, with grayscale values representing signal intensity. (*B*) Quantification of gal9 fluorescence intensity at the immune synapse or the rear of WT DCs in permeabilized (intracellular) and nonpermeabilized (surface) samples (n = 4 to 6 donors, 6 to 10 cells analyzed per donor). (*C*) Volcano plot depicting gal9-interacting proteins identified by mass-spectrometry following immunoprecipitation with α-gal9 (*Right*) or α-IgG (*Left*). MHC-II (HLA-DRB4) molecule is highlighted in red and gal9 (*lgals9*) in pink. Dotted gray lines depict the cut-off values; the fold change (x-axis) of 2 and *P*-value (y-axis) of 0.05. Black dots represent proteins that had fold change >2 and *P*-value >0.05. (*D*) Immunoprecipitation (IP) of untreated or lactose-treated (35 mM) DCs using α-HLA-DR antibodies or isotype as negative control and resolved and probed with α-gal9 and α-HLA-DR antibodies (n = 3). (*E*) Schematic representation of the NMR experimental design. Gal9 was incubated with HLA-DR α and/or β chain intracellular regions and chemical shift perturbation measurements performed on gal9 amino acids. Lactose (LacNAc) was used as glycan binding control. (*F*) Selected ^1^H,^15^N-HSQC NMR spectrum of ^15^N-gal9-alone (black) incubated with 1:100 (orange) or 1:200 (purple) of the intracellular domain of HLA-DR β chain. (*G*) Spectra of gal9 alone (black), with 200 equivalents of α and β HLA-DR intracellular domains (orange), and 300 equivalents of lactose (purple). Labels in each crosspeak indicate whether it belongs to gal9 N- or C-ter domains. Arrows indicate the direction of chemical shift perturbations upon peptide addition. All graphs show mean ± SEM. Statistical analysis was conducted by using ordinary one-way ANOVA with Tukey’s multiple comparisons (*B*) or Friedman test (*D*). ns *P* > 0.05, *P < 0.05, ***P < 0.001, ****P < 0.0001.

To mechanistically resolve how gal9 regulates IS formation we examined whether gal9 localized to the contact zone between DCs and T cells using airyscan confocal microscopy. Intracellularly, gal9 was distributed uniformly in naïve DCs but was recruited to the IS upon T cell interaction (Fig. 4 *A* and *B* and *SI Appendix*, Fig. S7 *A* and *B*). Interestingly, we failed to detect specific recruitment of surface gal9 to the IS upon T cell engagement (Fig. 4*B* and *SI Appendix*, Fig. S7 *A* and *B*) confirming a role for intracellular gal9 in IS assembly.

Next, we performed immunoprecipitation coupled with mass spectrometry (IP-MS) to identify gal9 binding partners in DCs. Gal9 was significantly enriched in the gal9-IP compared to isotype control, confirming the quality of the pull-down (Fig. 4*C*). To account for donor variability, we performed two independent IP-MS with DCs isolated from six independent donors (*SI Appendix*, Fig. S7*C*). Forty binding partners were enriched at least 1.5-fold in gal9-IP compared to isotype control in both IP-MS experiments, including previously reported interactions (e.g., CD44, Vamp3) (*SI Appendix*, Fig. S7*C*) (18). Analysis of gal9-binding proteins identified HLA-DR to interact with gal9 (Fig. 4*C* and *SI Appendix*, Fig. S7*C*). Gene Ontology (GO) analysis of gal9 binding partners showed a functional enrichment in MHC-class II and IS-related pathways (*SI Appendix*, Fig. S7*D*). HLA-DR-gal9 interaction was confirmed by coimmunoprecipitation followed by western blot (Fig. 4*D*). To test glycan dependency, co-IPs were performed on lactose-treated DCs using either an HLA-DR specific antibody or isotype control. Addition of lactose, a competitive inhibitor of glycan-based galectin interactions (19, 29), removed 70 to 80% of surface gal9 (*SI Appendix*, Fig. S7 *E* and *F*) but did not affect gal9 binding to HLA-DR (Fig. 4*D* and *SI Appendix*, Fig. S7*G*), indicating both proteins interact in a glycan-independent (intracellular) manner in DCs.

To confirm a direct interaction between HLA-DR and gal9 we turned to NMR and investigated gal9 binding to HLA-DR intracellular regions (Fig. 4*E*). ^15^N-labeled gal9 was titrated with increasing concentrations of either α or β HLA-DR intracellular peptides. ^1^H,^15^N HSQC gal9 spectrum was acquired at each titration point, allowing detection of chemical shift perturbations in specific gal9 amino acids. While most backbone amide crosspeaks were unaffected, a subset exhibited progressive chemical shift perturbations (Fig. 4*F* and *SI Appendix*, Fig. S8 *A*–*C*), consistent with specific protein–peptide interactions (30). As shown, residues from both N- and C-ter gal9 domains were perturbed upon incubation with either α or β HLA-DR intracellular tails, suggesting the participation of both gal9 domains in the molecular recognition of HLA-DR. In addition, crosspeak changes in gal9 residues persisted in the presence of lactose, indicating that the HLA-DR peptide binding site in gal9 is distinct from its two glycan binding sites (Fig. 4*G* and *SI Appendix*, Fig. S8*D*).

**Fig. 5.**
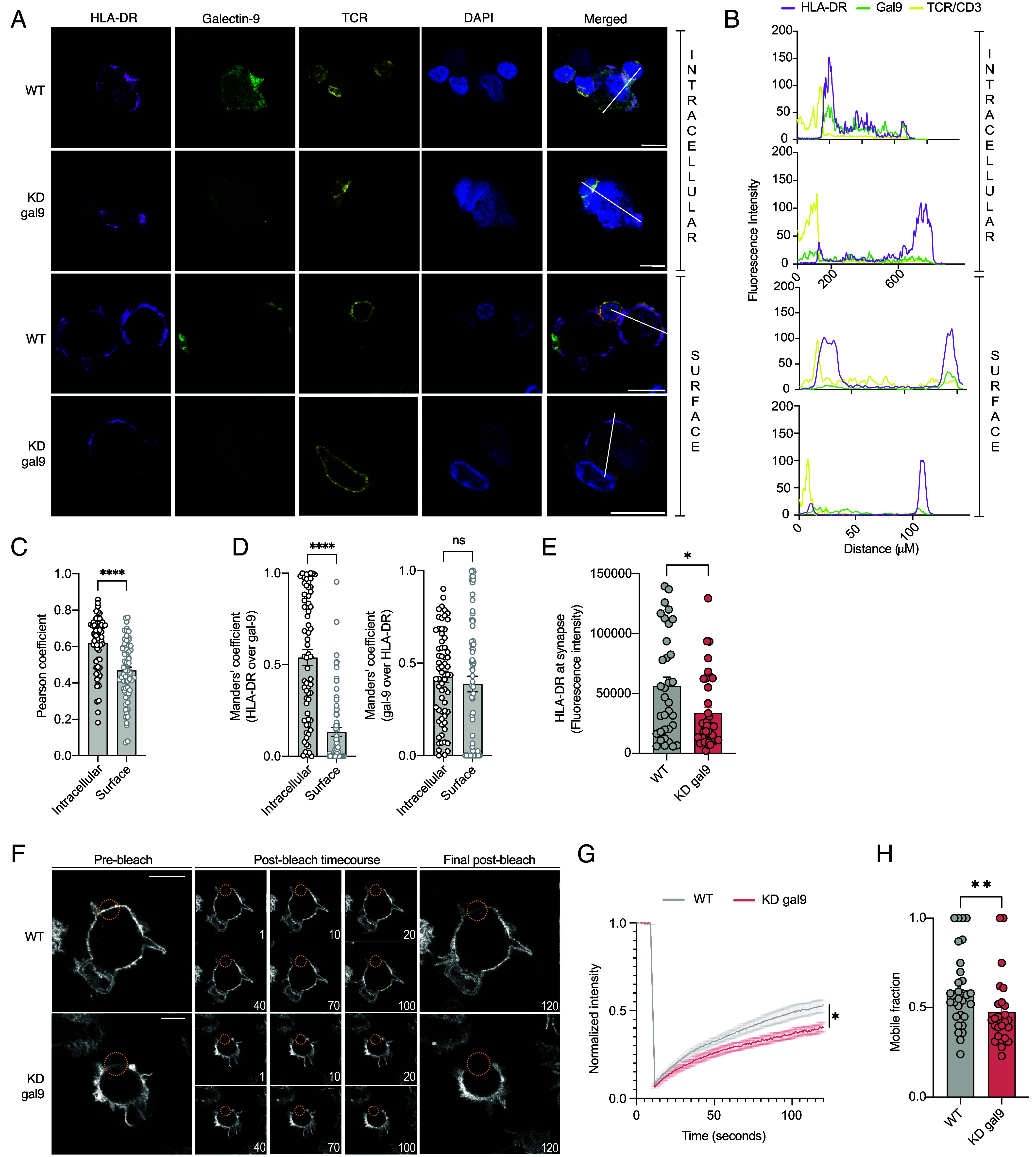
Intracellular gal9 regulates the recruitment of HLA-DR at the immunological synapse and its lateral mobility on the plasma membrane. (*A*) Representative airyscan confocal microscopy of WT or KD gal9 DCs incubated for 2 h with T cells and stained for HLA-DR, gal9, TCR/CD3, and DAPI. Permeabilized (intracellular) and nonpermeabilized (surface) conditions shown. (*B*) Line scans of fluorescence intensity across the synapse to rear (white line in merged composite in (*A*) of DCs depicting HLA-DR (magenta), gal9 (green), and TCR (yellow) signals. (*C* and *D*) Pearson’s (*C*) and Mander’s (*D*) correlation coefficient quantifying HLA-DR and gal9 colocalization in permeabilized (intracellular) and nonpermeabilized (surface) DCs (n = 4, 10 to 20 cells analyzed/donor). (*E*) Quantification of intracellular HLA-DR intensity at the synapse in WT and KD gal9 DCs cocultured with T cells (n = 4, 10 to 20 cells analyzed/donor). (*F*) Representative FRAP images showing HLA-DR recovery in WT and KD gal9 DCs. Images depict HLA-DR signal and distribution prior to bleaching (*Left*), during the postbleach recovery phase (middle), and at the final postbleach time point (*Right*). The bleached region is indicated by a yellow circle, with time points (in seconds) labeled in the figure. (*G*) Mean FRAP recovery curves of HLA-DR in WT (gray) vs KD gal9 DCs (red), normalized to prebleach intensity. (*H*) Quantification of HLA-DR mobile fractions in WT and KD gal9 DCs (n = 4 donors, 6 to 15 cells analyzed/donor). (Scale bar, 10 μm.) Each dot represents a differenT cell. Statistical analysis was performed by the Mann–Whitney test (*C*, *D*, and *H*), one-way ANOVA (*E*), Friedman test (*G*). ns *P* > 0.05, *P < 0.05, **P < 0.005, ****P < 0.0001.

HLA-DR is responsible for presenting antigens to CD4^+^ T cells and thus we further examined whether its interaction with gal9 underlies impaired IS formation in KD gal9 DCs. HLA-DR and cytosolic gal9 colocalized at the DC side of the IS (visualized by TCR localization) using airyscan confocal microscopy (Fig. 5 *A* and *B*), confirmed by Pearson’s (Fig. 5*C* and *SI Appendix*, Fig. S9*A*) and Mander’s (Fig. 5*D* and *SI Appendix*, Fig. S9*B*) correlation coefficients. This colocalization was only observed after permeabilization (intracellular) and could not be detected when examining surface-bound gal9, supporting that intracellular gal9 drives IS formation (Fig. 5 *A* and *B*). Gal9 depletion reduced HLA-DR recruitment to the IS (Fig. 5 *A*, *B*, and *E*), confirming a functional relationship between HLA-DR and gal9. Next, we assessed whether gal9 modulates the dynamic behavior of HLA-DR on the membrane using fluorescence recovery after photobleaching (FRAP) experiments on WT and KD gal9 DCs. HLA-DR exhibited limited fluorescence recovery rate with a lower proportion of HLA-DR molecules able to move back into the bleached area (mobile fraction) in KD gal9 DCs compared to WT (Fig. 5 *F*–*H* and *SI Appendix*, Fig. S9*C*). The rate at which HLA-DR fluorescence recovered to 50% of its final intensity (T-half) was similar between KD gal9 and WT DCs (*SI Appendix*, Fig. S9*D*), indicating that gal9 influences the fraction of mobile HLA-DR molecules, but not its diffusion rate. Overall, these results demonstrate intracellular gal9 depletion disrupts HLA-DR recruitment to the IS and limits its mobility across the membrane, thus establishing intracellular gal9 as essential for synapse formation and subsequent T cell activation and proliferation.

### DCs From gal9 KO Mice Are Impaired in Tumor Rejection Capacity In Vivo.

**Fig. 6.**
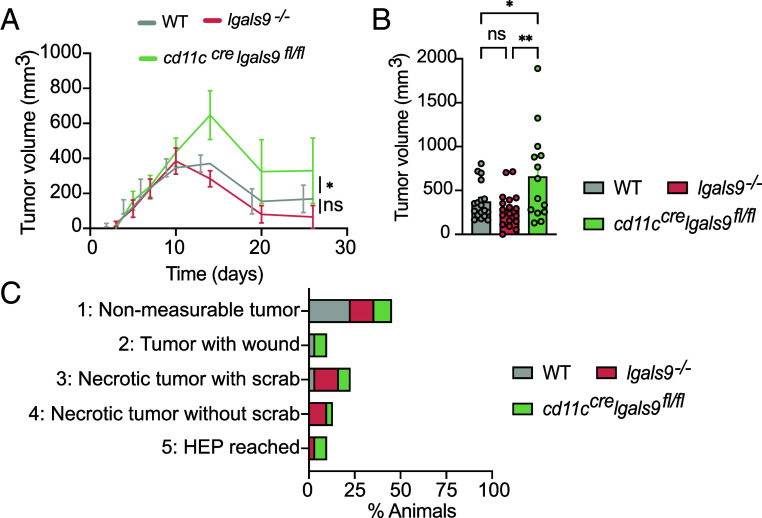
Gal9 loss in DCs impairs tumor rejection in vivo. (*A*) RMA-Muc1 tumor volume over time in WT (gray), *lgals9*^−/−^ (red), and *cd11c^cre^Lgals9^fl^*^/fl^ (green) (n = 14 to 18 mice/genotype). (*B*) Tumor volume at day 14 postinjection in the same mouse cohorts. (*C*) Distribution of animal discomfort levels (%) during tumor progression, assessed per institutional guidelines. Nonmeasurable tumors: Tumors present but too small to be measured with calipers. HEP: Humane end point. All data are shown as mean ± SEM. Statistical analysis was performed by one-way ANOVA with multiple comparisons (*A*), two-way ANOVA with Tukey’s test (*C*), and standard significance thresholds: ns *P* > 0.05, *P < 0.05, **P < 0.005.

Since gal9 is also expressed and plays important roles in other immune cells, we generated gal9 conditional knockout mice to specifically dissect the effects of gal9 in DCs in vivo. *Lgals9*-floxed (*lgals9^fl/fl^*) mice were crossed with *cd11c^cre^* transgenic mice to generate DC-gal9 conditional KO mice (*cd11c^cre^lgals9^fl/fl^*). Characterization of the myeloid compartment of WT, *gal9^−/−^*, or *cd11c^cre^lgals9^fl/fl^* mice demonstrated no impairment in DC development upon gal9 loss (*SI Appendix*, Figs. S10 and S11). Similar numbers of conventional DC type 1 (cDC1), plasmacytoid DC or conventional DC type 2 (cDC2) were observed in lymph organs of WT, *gal9^−/−^*, or *cd11c^cre^lgals9^fl/fl^* mice (*SI Appendix*, Fig. S11 *A* and *B*), and migratory cDC1 and cDC2 subsets were also found in comparable numbers (*SI Appendix*, Fig. S11 *C* and *D*). In addition, no differences in the percentage of macrophages, eosinophils, neutrophils, or monocytes were observed between WT, *gal9^−/−^*, or *cd11c^cre^lgals9^fl/fl^* mice (*SI Appendix*, Fig. S11 *E*–*H*), implying that gal9 is not required for myeloid cell development and differentiation. Next, we investigated the T cell–dependent tumor rejection model RMA-Muc1 (31) to determine whether gal9 expression in DCs was relevant to T cell function in vivo. Loss of gal9 in DCs delayed antitumor responses as tumor burden in *cd11c^cre^lgals9^fl/fl^* mice was larger as compared to WT and *gal9*^−/−^ mice after day 14 and until day 25 post tumor inoculation (Fig. 6 *A* and *B*). Gal9 conditional knockout mice exhibited more severe discomfort stages compared to WT and *gal9* ^−/−^ strains. Notably, >15% of *cd11c^cre^lgals9^fl/fl^* mice reached humane endpoints (HEP) and suffered from high-discomfort levels, whereas none of the WT mice did, highlighting the impact of gal9 deficiency on disease progression (Fig. 6*C*). These data indicate that gal9 expression in DCs controls T cell antitumor activity in vivo.

## Discussion

DCs are crucial for initiating cellular immunity by activating T cells through MHC-mediated antigen presentation, a process dependent on the formation of an IS (1). While the molecular mechanisms on T cells are well studied, how IS components traffic and assemble at the plasma membrane of DCs remains elusive.

Here, we identified gal9 is required for IS formation and describe a functional interaction with the cytosolic α and β domains of MHC-II (HLA-DR). Gal9 depletion in DCs disrupted HLA-DR membrane lateral mobility as well as its recruitment to the IS upon T cell binding, hindering stable DC-T cell interactions, which ultimately impaired CD4^+^ T cell proliferation. In vivo, the absence of gal9 in DCs resulted in increased tumor growth, underlining its importance in DC-mediated immune responses. Our findings demonstrate that gal9 enables sustained DC-T cell contacts and effective CD4^+^ T cell proliferation (*SI Appendix*, Fig. S12).

Contrary to its immunosuppressor functions in lymphocytes (32–34), we and others have shown Gal9 stimulates DC function (17–19). Data presented here highlight a previously unreported role for gal9 driving DC-mediated T cell activation, key to launch cellular adaptive immunity. Our findings are in line with recent single cell RNAseq data showing low gal9 expression in intratumoral murine DCs correlated with fewer T cell interactions (35). Moreover, our newly generated DC conditional gal9-KO mice exhibited increased tumor growth compared to both full gal9 KO and WT counterparts, underscoring the critical role of gal9 in T cell–mediated antitumor immunity. The discrepancy between conditional and total gal9 KO mice in rejecting the tumor may be explained by the cell specific anti/proinflammatory functions of gal9, due to the distinct binding partners expressed by different immune cells.

While galectins have been shown to mediate molecular events at the T cell side of the IS (21–23, 36), their roles in DCs at the IS remain poorly characterized. Galectin-1 (gal1) and -3 depletion enhanced T cell activation (37, 38), likely reflecting differential galectin binding partners and activation of downstream signaling pathways. On the other hand, extracellular galectin-8 (gal8) has been implicated in modulating DC membrane dynamics and enhancing antigen presentation via MHC-II (36). Gal8 is a tandem-repeat galectin similar to gal9, and could putatively bind MHC-II extracellularly through its conserved N-glycosylation sites (N78, N118, and N19 in humans; N78, N118, and N23 in mice) (39). At the synaptic extracellular interface, galectin-mediated interactions through N-glycans could also support cis-interactions, ensuring proper TCR-MHC engagement and distribution (20, 40). Although we cannot disregard an extracellular interaction between gal9 and HLA-DR, our data demonstrate that gal9 in DCs regulates IS formation intracellularly. Coimmunoprecipitation of HLA-DR in lactose-treated DCs showed only a minor reduction in gal9 binding, indicating that most gal9–HLA-DR interactions are glycan-independent and thus likely occur inside the cell. This is supported by NMR measurements and airyscan microscopy where gal9 was absent at the DC-T cell IS interface in nonpermeabilized cells but clearly detectable intracellularly. Remarkably, HLA-DR membrane expression and upregulation upon maturation were not altered in response to gal9 depletion, suggesting gal9 is not required for HLA-DR expression or translocation to the membrane. This is in line with recent literature showing that the secretion pathways for MHC-II and gal9 differ fundamentally in their mechanisms and molecular machinery. MHC-II traffics via the conventional endosomal secretory pathway (41) and gal9 secretion involves Tim-3 (42), LAMP2 (43), and ATG9A (44), underscoring a unique and highly regulated pathway distinct from conventional secretory mechanisms. Inhibition of gal9 secretion machinery and thus blocking its surface expression could offer further insights into the crosstalk of surface and gal9 intracellular pools in IS formation.

In addition, we observed no differences in cathepsin expression or activity within endocytic compartments in human or murine gal9-depleted DCs compared to their WT counterparts. Given that cathepsins, particularly Cathepsin S, are crucial for the HLA-DR maturation (45, 46), our findings indicate that gal9 does not influence antigen processing or peptide loading in the endocytic compartment. Although RNA-seq analysis indicated a modest upregulation of MHC-II-related pathways following gal9 loss, no corresponding differences were observed at the protein level in antigen processing or presentation via MHC-I or -II. Thus, the intracellular role of gal9 in organizing HLA-DR at the IS appears to be independent of any potential function in antigen processing.

In a resting state, newly synthesized peptide–MHC-II complexes are delivered to the DC surface in <100 nm microclusters (47). These complexes exhibit a dynamic spatial organization, transitioning from a constitutively clustered, lipid raft-associated, configuration to a highly ordered arrangement at the IS (48). Upon T cell engagement, MHC-II molecules rapidly accumulate at the contact interface. Lipid rafts and tetraspanins (e.g., CD9 and CD81) play pivotal roles in this process, by acting as signaling platforms and enhancing antigen presentation efficiency (49–51) Extrinsic factors such as ICAM-3–LFA-1 interactions initiate IS formation and promote MHC-II clustering via the Vav1–Rac1–actin axis (6). Although actin remodeling does not directly regulate MHC-II dynamics (52), mutations in actin regulators like WASp impair MHC-II polarization indicating that actin also exerts essential effects on IS stability (10). We previously demonstrated that gal9 modulates RhoA and Rac1 activity to maintain cortical actin dynamics (19), suggesting that gal9 could impact MHC-II membrane positioning by regulating the actin cytoskeleton. In parallel, lipid raft-associated mechanisms also contribute to MHC-II organization. Flotillin-1 and -2 (flot1/2), key components of cholesterol-rich membrane domains, facilitate antigen presentation (49, 53). Our IP-MS data highlighted Flot1/2 as putative gal9 binding partners, suggesting that gal9 may stabilize membrane microdomains essential for synapse integrity beyond its effects on MHC-II. Our data showing gal9 directly interacts with the intracellular tails of MHC-II allows us to hypothesize that gal9 may act as a molecular bridge, anchoring MHC-II molecules to cytoskeletal and membrane-organizing machinery. Consistent with this, our FRAP analysis demonstrated that gal9 promotes the proportion of mobile HLA-DR molecules, without affecting the diffusion rate of those that remain immobile. These findings support a model in which gal9 functions as a stabilizing scaffold, coordinating both actin dynamics and membrane domain integrity to regulate MHC-II positioning at the ISIn summary, here we describe an intracellular function for gal9 in organizing MHC-II recruitment and localization at the IS, providing insights into how galectins regulate immune receptor positioning to enhance T cell activation. Data presented here highlight a role for gal9 in promoting DC function, underscoring the broad impact of galectins in regulating immune responses.

## Materials and Methods

### Generation of Monocyte-Derived DCs.

Monocyte-derived DCs (DCs) were derived from peripheral blood monocytes isolated from buffy coats (Sanquin Bloedvoorziening, Nijmegen, informed consent obtained) using CD14+ beads (following the manufacturer’s instructions, #130-050-201, Miltenyi). Monocytes were cultured up to five days in RPMI 1640 medium (Life Technologies) containing 10% fetal bovine serum (FBS, Greiner Bio-one), 1 mM ultra-glutamine (BioWhittaker), antibiotics (100 U/ml penicillin, 100 µg/ml streptomycin and 0.25 µg/ml amphotericin B, Life Technologies), IL-4 (500 U/ml, Miltenyi), and GM-CSF (800 U/ml, #130-093-868, Miltenyi) in a humidified, 5% CO_2_. On day 3, medium was refreshed with new IL-4 (500 U/ml) and GM-CSF (800 U/ml). On day 6, moDCs were supplemented with a maturation cocktail: IL-6 (15 ng/ml, #130-093-933, Miltenyi), TNF-α (10 ng/mg, #130-094-014 Miltenyi), IL-1β (5 ng/ml, #130-093-898 Miltenyi) and PGE2 (10 µg/ml, Pfizer).

### Mice.

The conditional *Lgals9* mouse strain (MGI:6466637) was generated on the C57Bl/6JRj background using pronuclear microinjection in mouse zygotes. The injection mixture consisted of water with 200 ng/µl Cas9 protein (IDT), two sgRNA (25 ng/µl each) targeting the intronic sequence flanking exon 3 of *Lgals9* (5’-CGGGACTAGAGCGTGTCTTAGGG-3’ and 5’-TAAAACCCAGCGGGCGAATGGGG-3’) and 15 ng/µl of a long single-stranded DNA oligo containing exon 3 of *Lgals9* flanked by two loxP recombination sites and homology arms. The hom floxed mice were sequence verified.

Sex- and age-matched C57Bl/6 J WT (*lgals9^+/+^*), and *lgals9^−/−^* littermate mice were bred at the Central Animal Laboratory, Nijmegen. Both male and female age-matched mice were used across replicate experiments. OT-I and OT-II mice were sourced from Charles River. All mice were housed in top-filter cages, provided a standard diet, and had unrestricted access to water and food. Mice were used at ages ranging from 6 to 18 wk. All murine studies complied with European legislation (directive 2010/63/EU of the European Commission) and were approved by local authorities (CCD, The Hague, Netherlands) for the care and use of animals with related codes of practice.

### Isolation and Culture of Primary Cells.

T cells were isolated from peripheral blood mononuclear cells derived from healthy individuals (Sanquin) using the Pan T cell isolation kit (#130-096-535, Miltenyi) and the Pan Naïve T cell kit (#130-097-095, Miltenyi) according to the manufacturer’s instructions. After isolation, T cells were cultured in X-VIVO-15 (Lonza) supplemented with 2% human serum (HS, Sigma-Aldrich).

For murine DCs, lymph nodes and spleens from wild-type C57BL/6 J, *lgals9^−/−^*, *cd11c^cre^lgals^fl/fl^* mice were isolated and meshed through a 100 μm cell strainer to obtain single cell suspension. Cells were spun at 400xg for 5 min, resuspended for 5 min at room temperature (RT) in 1.5 ml of 1x ammonium chloride potassium (ACK) for erythrocytes lysis and washed twice with PBS (20 ml). DCs were isolated using the CD11c MicroBeads UltraPure isolation kit (#130-125-835, Miltenyi).

### Small Interfering RNA Knockdown.

On day 3 after isolation, DCs were harvested and subjected to electroporation. Three custom stealth small interfering RNA (siRNA) were used to silence galectin-9 (LGALS9HSS142807, LGALS9HSS142808, and LGALS9HSS142809) (Invitrogen). Equal amounts of the siRNA ON-TARGETplus nontargeting (here referred to as wildtype) siRNA#1 (Thermo Scientific) were used as control. Cells were washed twice in PBS and once in OptiMEM without phenol red (Invitrogen). A total of 15 μg siRNA (5 μg from each siRNA) was transferred to a 4-mm cuvette (Bio-Rad). 5 to 10 × 10^6^ DCs were added in 200 μl OptiMEM and incubated for 3 min before being pulsed with an exponential decay pulse at 300 V, 150mF, in a Genepulser Xcell (Bio-Rad). Immediately after electroporation, cells were transferred to preheated (37 °C) phenol red–free RPMI 1640 supplemented with 1% ultraglutamine, 10% (v/v) FCS, IL-4 (300 U/ml), and GM-CSF (450 U/ml) and seeded at a density of 5x10^5^ cells/ml.

### Flow Cytometry.

To determine galectin-9 depletion following siRNA transfection, single cell suspensions were incubated with 2% HS for 10 min on ice to block nonspecific interaction prior to being stained with a goat anti-galectin-9 antibody (#AF2045, R&D systems) at 8 μg/ml for 30 min at 4 °C. A donkey-anti-goat secondary antibody conjugated to Alexa Fluor 488 or 647 was used (Invitrogen; 1:400 (v/v)).

To determine T cell activation or proliferation, harvested cells were blocked in PBA buffer containing 2% HS for 10 min. Afterward, T cells were stained in 2% HS PBA (0.1% BSA, 0.01% NaH_3_ in PBS) with the relevant antibodies (*SI Appendix*, Table S1) at 1:25 (v/v) for 20 min.

Splenic and lymph node-cell suspensions from C57Bl/6 J WT (*lgals9^+/+^*), *lgals9^−/−^*, and *cd11c^cre^lgals^fl/fl^* animals were blocked with 5% mouse serum for 10 min on ice prior to being incubated with the corresponding antibody mixture (DC subset and myeloid panels- *SI Appendix*, Table S1, 30 min at 4 °C). Cells were then washed twice and incubated with Brilliant Violet 605™ Streptavidin [#405229, Biolegend, 1:100 (v/v)] for 15 min at 4 °C. Live/dead staining was performed with eFluor780 or zombie violet viability dyes (1:2,000, ThermoFisher) in PBS for 20 min at 4 °C after which cells were fixed in 4% PFA for 10 min at RT.

Data were acquired using a FACSVerse or a FACSLyric instrument (BD, Frankllin lakes, NJ) and analyzed using the Cytobank platform (Beckman Coulter Life Sciences) (54).

### MLR.

Allogeneic Pan T cells were stained with CFSE or CellTrace™ VioleT cell Proliferation Kit (C34554 or C34557, 5 μM, Life Technologies) for 30 min at 37 °C. The reaction was stopped by blocking with 1:1 (v/v) FBS 5 to 10 min at RT. Cells were washed extensively and counted. WT or galectin-9 knockdown DCs were cultured with CTV/CFSE-labeled T cells in a 1:10 ratio for 6 d at 37 °C, 5% CO_2_. Afterward, T cells were measured on the flow cytometer to evaluate the loss of cell trace dye expression. When needed, galectin-9 knockdown DCs were incubated with 1 µg/ml human recombinant galectin-9 protein (R&D systems, #2045-GA-050) for 30 min prior to being cocultured with T cells. Alternatively, DCs were treated with 10 µg/ml galectin-9 blocking monoclonal antibody (Merck Millipore, #MABT834, clone 9S2).

The proliferation index (PI) was used to quantify the number of times an original T cell divided [(PI)/2], where PI was the percentage of cells in each peak of division.

### Autologous T cell Activation Assays (Mouse and Human).

To assess MHC-II-mediated antigen presentation, day 6 WT or galectin-9 depleted moDCs were cultured with 0.5 μg/ml TT (Sigma Aldrich; #582231) for 2 h. Loaded DCs were washed twice with PBS and cocultured with autologous CTV-labeled CD4^+^ T cells at 1:25-50 ratio (DC:T cell) for 6 d prior to cells being harvested and T cell proliferation determined by flow cytometry.

To assess MHC-I-mediated antigen presentation, day 6 HLA-A*0201-type WT or galectin-9 knockdown moDCs were seeded at 1 × 10^6^ cells/ml and treated with 1 μg/mL LPS (Invivogen, #vac-3pelps) together with 1 μM of gp-100 peptide (synthesized by Genscript) or 1 μM (irrelevant) NY-ESO1 peptide (SLLMWITQC; synthesized by Genscript) for 2 h at 37 °C, 5% CO_2_. Afterward, cells were washed and seeded at the aforementioned density in X-VIVO media supplemented with 2% HS for 2 h at 37 °C. Autologous CD8^+^ T cells were washed in PBS, resuspended in 250µL phenol red -free X-VIVO medium and transferred into a 4-mm cuvette (Bio-Rad). Directly prior to electroporation, 20 μg/10 × 10^6^ cells HLA-A2-gp100 TCR mRNA (provided by BioNtech) was added into the cell suspension. Cells were then pulsed with a square wave protocol at 500 V, 3 ms, 1 pulse in a Genepulser Xcell (Bio-Rad) and transferred for 1 h (37 °C, 5% CO_2_) into 1 mL red phenol-free X-VIVO medium (supplemented with 5% HS without antibiotics). moDCs were cocultured with autologous transfected T cells at a 1:5 ratio (DC:T cell) for 3 d prior to being harvested and T cell proliferation determined by flow cytometry. TCR transfection was confirmed using an MHC-Dextramer (HLA-A*0201/YLEPGPVTA) (Immudex)S4A.

Splenic OT-I or OT-II mouse T cells were isolated with the murine CD4^+^ and CD8^+^ T cell isolation kits (#130-104-454 and 130-104-075, Miltenyi). Wild type or *galectin-9*^−/−^ CD11c+ cells were treated for 2 h with 1 μg/mL LPS and primed with 25 or 75 µg/mL of OVA EndoFit™ (InvivoGen, #vac-pova) or 1 µg/mL OVA peptides (OVA_257-264_ for OT-I cells or OVA_323-339_ for OT-II cells, from InvivoGen, #vac-sin and #vac-isq, respectively). Primed DCs and OT-I or OT-II cells were cocultured at a 1:5 ratio for 3 d after which T cell proliferation was determined as before. When necessary, *galectin-9*^−/−^ DCs were incubated with 2 µg/ml mouse recombinant galectin-9 protein (R&D systems, #3535-GA-050) for 1 h prior to being cocultured with T cells.

### ELISA.

Enzyme-linked immunosorbent assay kits (Invitrogen, ThermoFisher Scientific) were used to measure IFN-γ (KIT REF: 88.7316.88) from supernatants obtained from DC-T cell cocultures. Supernatants were diluted 1:50 (for the 6-d cocultures) or 1:2 (for the 48 to 120 h cocultures). Protocols were performed following the manufacturer’s instructions.

### Coimmunoprecipitation, Western Blotting and Mass Spectrometry.

Untreated or lactose-treated (35 mM) (19) moDCs were harvested (1 × 10^6^ cells) and lysed in 100 µl of lysis buffer (1% brij-97, 10 mM Tris-HCl pH 7.5, 150 mM NaCl, 2 mM MgCl_2_, 2 mM CaCl_2_, protease inhibitor). Unspecific protein binding was precleared using isotype-coated Protein G Sepharose beads (#17-0618-01, Cytiva). Coimmunoprecipitation was performed by incubating samples in 3 µg of either mouse αIgG2aκ (#400202, Biolegend) or mouse αHLA-DR (#307602, Biolegend) for 1 h at 4 °C. Protein G Sepharose beads were used to precipitate protein–antibody complexes, followed by extensive washing, and elution in nonreducing 4× Laemmli Sample Buffer (#1610747, Bio-Rad). To elute immunoprecipitated proteins, beads were boiled for 5 min at 95 °C. For western blotting, samples were separated by 12% SDS-PAGE and transferred to PVDF membranes. Membranes were blocked in PBS Blocking Buffer (#927-70001, LI-COR), and incubated with 1 µg/ml mouse αHLA-DR (#327002, Biolegend) overnight at 4 °C and with 0,1 µg/ml secondary donkey α-mouse IRDye680 at RT. For gal9 probing, membranes were blocked in TBS Blocking Buffer (#927-60001, LI-COR) and probed with 1 µg/ml goat α-human gal9 (#AF2045, R&D Systems) overnight at 4 °C. After washing, 0,1 µg/ml secondary donkey α-goat IRDye800 was added to the membrane. IP and Co-IP were validated using the Amersham Typhoon 5 (Cytiva) in the IRlong and IRshort channels. Images were analyzed using Fiji and band intensity was quantified by measuring the integrated density.

For mass spectrometry analysis, immunoprecipitation on DCs was performed as previously described (18).

### ^15^N-Gal9 Expression and Purification and ^1^H-^15^N Heteronuclear Single Quantum Coherence (HSQC) Spectroscopy.

The full-length short isoform of human Galectin-9 (amino acids 1 to 311) was cloned into a pET3a vector, expressed in *E. coli* BL21 cells using ^15^N-labeled ammonium chloride in M9 medium, and purified via lactose affinity and size exclusion chromatography. Purity was confirmed by SDS-PAGE and LC-MS, and lactose was removed through dialysis and centrifugal filtration, with NMR confirming its absence. For NMR, ^15^N-Gal9 was prepared at 14 μM in phosphate buffer and analyzed using ^1^H-^15^N HSQC spectroscopy on a Bruker 800 MHz spectrometer. Peptides were added at a 100-fold molar excess, and chemical shift perturbations were monitored to identify residue-specific interactions. Data analysis included comparison of apo and peptide-bound spectra to map binding sites across both gal9 domains. See *SI Appendix*, *Supplementary Materials and Methods* for full details.

### Immunofluorescence and Confocal Microscopy.

Day 6 DCs were stimulated with 1 µg/mL Staphylococcal enterotoxin B for 1 h at 37 °C, followed by thorough washing. Mature wild-type or gal9 knockdown DCs were cocultured with autologous T cells (1:2 ratio) for 2 h, then transferred to PLL-coated coverslips, adhered for 10 min, and fixed with 4% PFA. After quenching with NH_4_Cl, samples were either permeabilized for intracellular staining or left intact for surface staining, then incubated overnight at 4 °C with anti-gal9 antibody. The next day, cells were stained with fluorescent antibodies targeting T cell markers and HLA molecules, followed by DAPI nuclear staining and mounting in Mowiol. Imaging was performed using both Leica DMI6000 and Zeiss LSM900 microscopes, and fluorescence analysis was conducted in Fiji ImageJ using custom macros and statistical tools like Mander’s and Pearson’s coefficients. See *SI Appendix*, *Supplementary Materials and Methods* for full details.

### Live Cell Microscopy.

Day 6 moDCs were treated with superantigen B as previously stated. Before coculturing and to enable cell segmentation during data analysis, DCs were stained with FarRed Cell Trace™ dye (#C34564, Thermo Fisher) according to the manufacturer’s instructions and autologous T cells were stained with CFSE Cell Trace™ dye (#C34554, Thermo Fisher). Both cell types were seeded on a black 96-well, f-bottom microplate (#655076, Greiner) and immediately acquired.

Time-lapsed video microscopy was performed using the Celldiscoverer7 (Zeiss), using the 20x objective with 0.5x tube lens or the BD Pathway 855 spinning disk confocal microscope (BD Bioscience), the atto vision software (BD Bioscience) and the 10X objective (Olympus). Sequential images were acquired every 3 min for 2 h using the 555/30 excitation filter (Chroma), 631/33 excitation filter (Chroma), emission filter 84101 (Chroma), and dichroic filter 84000 (Chroma). Time-lapse sequences were analyzed with the Trak Mate plugin (Fiji) to measure cell velocity.

### RNAseq.

Total RNA was extracted from mature moDC using zymo quick-RNA miniprep (#R1055) according to manufacturer instructions and used as input for library preparation using the KAPA RNA HyperPrep Kit (#KR1351, KAPA BIOSYSTEMS). The resulting library was sequenced paired-end on an Illumina Nextseq 500 platform. Reads were aligned to the hg38 human genome using the seq2science pipeline (55), with STAR used as aligner. Reads that mapped equally well to multiple locations were discarded. Count normalization was performed using rlog normalization via DESeq2 in R (56). Differential genes were considered with adjusted *P*-value < 0.05 and a fold change > 1.5. For Gene Set Enrichment Analysis, fold changes per gene were determined by calculating the ratio between control and knockdown animals for each biological replicate separately. The average fold change was then used as input for the R package fgsea (57).

### Cathepsin and Proteasome Labeling.

Cathepsin and proteasome labeling was performed as described before (58). For cathepsin labeling, WT or KD gal9 DCs or isolated WT or KO gal9 CD11c^+^ splenocytes were harvested and resuspended at 10^6^ cells/ml. Aliquots of 10 µL (0.1^6^ cells) were treated with 0.5 µM of the BMV109 cathepsin probe and incubated for 1 h at 37 °C. Cells were centrifuged (10,000 rcf, 1 min, RT), resuspended in 9 µl hypotonic lysis buffer [50 mM PIPES (pH 7.4), 10 mM KCl, 5 mM MgCl_2_, 2 mM EDTA, 4 mM DTT, and 1% NP-40] and incubated 15 min on ice. Afterward, lysates were centrifuged (21,130 rcf, 15 min, 4 °C) and supernatant was transferred to fresh tubes. For proteasome labeling, 100,000 DCs or isolated CD11c^+^ splenocytes were spun down (10,000 rcf, 1 min, RT) and lysed in 9 µl hypotonic lysis buffer for 15 min on ice. Afterward, lysates were centrifuged (21,130 rcf, 15 min, 4 °C) and supernatant was transferred to fresh tubes. Lysates were treated with 1 µM MV151 and incubated for 1 h at 37 °C. All samples were diluted with 4× Laemmli sample buffer, denatured at 95 °C for 10 min, and analyzed using 15% SDS-PAGE analysis. Probe signal was visualized using an Amersham Typhoon 5, gel, and blot imaging system (Cytiva), followed by equal protein loading confirmation using Trypan Blue staining. Gel analysis was performed using Fiji ImageJ.

### DQ-OVA Endocytosis Assay and OVA-Presentation Assessment.

DQ^TM^-OVA (#D12053, ThermoFisher) was used as a substrate for assessing DC proteolytic activity. To ensure both WT and gal9 KD moDCs were exposed to the same concentration of substrate, one of the two was stained with Cell Trace Violet™ (#C34571, ThermoFisher, 1:2,000) and mixed in the same well at a 1:1 (WT:KD gal9) ratio of 1 × 10^5^ cells/well. Then, DCs were incubated with 200 nM Bafilomycin A1 (Sigma-Aldrich) and 0, 5, 10, or 20 μg/mL DQ-OVA for 1.5 h. After incubation, cells were washed extensively and incubated with 1 µg/ml of propidium iodide (in PBS) to differentiate between live and dead cells. Cells were measured using the MACSQuant Analyzer 10 Flow Cytometer (Miltenyi Biotec).

For murine DCs, the antigen processing capacity was determined by incubating the DCs for 24 h with 1 μg/ml LPS and 0, 2.5, or 5 mg/mL OVA (or 400 nM OVA_257-264_ peptide positive control). On the next day, the cells were blocked with 5% mouse serum, incubated with 1 ug/mL of propidium iodide and stained with CD11c (#117311, Biolegend; clone N418, af488-labeled, at 1:100) and H-2 Kb bound to SIINFEKL antibodies (#141606, Biolegend;clone 25-D1.16, APC-labeled, at 1:50). Stained cells were analyzed by flow cytometry using a FACSVerse.

### Fluorescence Recovery After Photobleaching (FRAP) Analysis.

Mature DCs (WT and gal9 KD) were blocked (5% HS) and stained with 10 μg/mL HLA-DR antibody (#307620, Biolegend, clone L243, af488-labeled) for 30 min on ice. Cells were then washed and seeded onto Willco dishes with phenol red-free Leibovitz media (#21083027, ThermoFisher) for 15 to 20 min at 37 °C prior to imaging. FRAP was performed using a Leica TCS SP8 microscope equipped for single-molecule detection, a 60× water immersion objective (1.2 NA), and an argon-ion laser set to 100% power at 488 nm for photobleaching. Fluorescence intensity was measured within the bleached region, across the entire cell, and in the background to correct for general photobleaching and background noise. Images were taken every 10 iterations of 1 s before bleaching, 4 iterations of 0.19 s during the bleach and 120 iterations of 0.19 s after bleaching. Mobile fractions were calculated manually and fluorescence recovery curves and recovery half-times (T½) were calculated using the easyFRAP online analysis tool (59).

### RMA-Muc1 Tumor Rejection Model.

*WT, lgals9^−/−^*, and *cd11c^cre^lgals^fl/fl^* female animals were injected subcutaneously with 5 × 10^6^ RMA-Muc1 cells (31). Tumor growth was monitored by measuring three dimensions—height (h), width (w), and length (l)—of each tumor using calipers every 2 to 3 d. Tumor volume was then calculated using the formula: π/6*w*l*h (mm^3^). Discomfort levels were evaluated by the animal caretakers and assesses based on the animal facility guidelines.

### MHC-II Immunopeptidomics.

All chemicals were from Sigma-Aldrich (analytical grade), unless mentioned otherwise. LC-grade water, methanol, and acetonitrile were from Biosolve. DCs were lysed in 100μL of 0.25% sodium deoxycholate, 1 mM iodoacetamide (Cytiva), 1 mM EDTA, 1% octyl-β-d-glucopyranoside (O3757-5 mL, Sigma), 1× cOmplete Protease inhibitor (#11836145001, Roche) and 0.1 mM phenylmethylsulfonyl fluoride (#93482-250 mL-F, Roche) on ice for 1 h. Lysates were cleared by centrifugation at maximum speed for 45 to 60 min at 4 °C. 5μL of Protein G Mag SepharoseTM (Cytiva) beads, prewashed with the lysis buffer, and 50 μg of anti-HLA-II (IVA-12) were added to the supernatant overnight at 4 °C under rotation after which beads-antibody-peptide-HLA complexes were washed 3 times with washing buffer (150 mM NaCl, 50 mM Tris-HCl pH 7.5). Peptides were eluted using 1% trifluoroacetic acid (TFA) (Uvasol® for spectroscopy, Merck) and desalted using custom-made 1 mL StageTips as previously described (60).

The StageTips were activated with 100µL methanol, washed with 100 µL buffer B [80% acetonitrile, 20% water, 0.1% formic acid (Honeywell)] and equilibrated with 100 µL buffer A (0.1% formic acid in water) twice by centrifuging the StageTips at 1,500×*g* for 1 min in custom-made 15 mL collection tubes. Samples were loaded onto StageTips, centrifuged at 1,000×*g* for 5 min until all of the sample passed through the C18 resin, washed once with buffer A at 1,500×*g* for 1 min and stored at 4 °C. Immunopeptidomes from WT or KD gal9 DCs were measured by single-shot LC-MS/MS on the Orbitrap Astral mass spectrometer (Thermo Scientific) connected to a Vanquish Neo nano-LC system (Thermo Scientific). For full details, see *SI Appendix*, Supplementary Materials and Methods.

### Statistical Analysis.

Pathway enrichment analysis was conducted using the gProfiler tool (version *e111_eg58_p18_f463989d*). The curated protein list was uploaded to gProfiler, where gene identifiers were matched to their corresponding pathways. The analysis focused on biological processes (dataset: GO terms or Reactome) and molecular functions (dataset: GO terms) relevant to the proteins of interest. The analysis utilized default settings, including statistical significance thresholds set at a *P*-value < 0.05. Enrichment results were interpreted using Benjamini–Hochberg correction for multiple testing. Pathways were considered significantly enriched if they met the specified thresholds. The results were visualized using R to illustrate the relationships and significance of the identified pathways.

All data were processed using Excel (Microsoft) and plotted using GraphPad Prism 8 or 10 (Version 8.0.2. or 10.0.0 GraphPad Software). Unless otherwise stated, all data are expressed as mean +/- SEM. The statistical test used to analyze each dataset is described in the corresponding figure legend. Data distribution was assessed using the Shapiro–Wilk’s test. Statistical significance was considered for *P* < 0.05.

## Supplementary Material

Appendix 01 (PDF)

Movie S1. **Live cell imaging movies of dendritic cell (DC) and T cell cocultures**. Wild-type (WT) DCs (magenta) interacting with T cells (green).

Movie S2. **Live cell imaging movies of dendritic cell (DC) and T cell cocultures**. Gal9 KD DCs (magenta) interacting with T cells (green). Time progression is indicated by the yellow timestamp (in minutes).

## Data Availability

Mass spectrometry dataset is deposited in the public proteomics identifications database PRIDE (dataset identifier PXD039781) (61). All sequencing data have been submitted to GEO under accession number GSE282411 (62). Study data are included in the article and/or supporting information.
