# Supplementary material for: Galectin-9 binding to HLA-DR in dendritic cells controls immune synapse formation and T cell proliferation
Source: Proc Natl Acad Sci U S A. 2025 Dec 8;122(50):e2501381122. doi: 10.1073/pnas.2501381122 (PMC12718305; doi:10.1073/pnas.2501381122)
Supplement: Supplementary file 1 — Appendix 01 (PDF) [file pnas.2501381122.sapp.pdf]

PNAS

Supporting Information for
Galectin-9 binding to HLA-DR in dendritic cells controls immune synapse formation and T cell proliferation

Andrea Rodgers-Furones[1], Thijs Brands[1,*], Guusje van Gameren[1,*], Mirane Florencio[2], Mayukha Bathini[3], Sandra Delgado[2], Zacharias Wijfjes[1], Kristina Fedorova[1], René Classens[1], Lona Kroese[4], Martijn Verdoes[1,5], Guido van Mierlo[1], Jesús Jiménez-Barbero[2,6,7,8], Rik G.H. Lindeboom[3], Ana Ardá[2,6] Annemiek B van Spriel[1], Laia Querol Cano[1,#].

Laia Querol Cano
Email: Laia.QuerolCano@radboudumc.nl

This PDF file includes:

Supporting text
Figures S1 to S12
Tables S1
Legends for Movies S1 to S2
SI References

Other supporting materials for this manuscript include the following:

Movies S1 to S2

Supporting Information Text

Supplementary Materials and Methods

15N-Gal9 expression and purification

The DNA fragment coding for the Galectin-9 full-length (short isoform, O00182-3 (UniProt), amino acids 1–311) was inserted into the pET3a (#RDB08416, provided by RIKEN BRC through the National BioResource Project of MEXT, Japan) expression vector, amplified in E. coli DH5α cells, and transformed into E. coli BL21 cells. A single colony was inoculated into 5 ml of LB and cultured for 6 h at 37°C. The preculture was centrifuged and resuspended in 1 ml of M9 medium, transferred to 200 ml of M9 medium, and incubated overnight at 37°C. A precise quantity of the culture was then added to 2 L of fresh M9 medium containing 1g/L 15NH4Cl. The culture was harvested by centrifugation at 6,000 rpm and pellet resuspended in lysis buffer (10mM Tris, pH 8, 300mM NaCl, 1mM phenylmethylsulfonyl fluoride) and sonicated at 4°C. The crude extract was clarified by centrifugation at 35,000 rpm for 1 h at 4°C. The soluble fraction was purified by α-Lactose-Agarose resin (Sigma-Aldrich) affinity chromatography and further purified by size exclusion chromatography in a SuperdexTM 75 Increase 10/300 column (Sigma, St. Louis, MO, United States). Gal9 purity was checked by 4–12% SDS-PAGE (Thermo Fischer, Waltham, MA, United States) and by LC-MS. To eliminate lactose from the protein sample, a series of dialysis and washes with centrifuge filters (Sartorius Vivaspin 6 10000 MWCO) using fresh buffer (PBS, 1mM DTT, 0.1% NaN3, pH7.4) were performed. The absence of lactose was confirmed by NMR.

1 H-15N Heteronuclear single quantum coherence (HSQC) spectroscopy

NMR samples (300µL total volume) were prepared in 5 mm Shigemi tubes. All NMR experiments, including 1H,15N-HSQC (heteronuclear single quantum coherence) spectra were acquired on a Bruker AVANCE II 800 MHz spectrometer equipped with a cryoprobe. Samples were prepared in 20mM sodium phosphate buffer containing 150mM NaCl at pH 7.4, using a solvent mixture of 90% H2O and 10% D2O. 15N-Gal9 was used at a final concentration of 14 µM. Peptides were initially added at a 200-fold molar excess relative to Gal9 from a stock solution of 6 mM in PBS, followed by dilution with additional 15N-Gal9 at 14 µM to achieve a final protein-to-peptide ratio of 1:100. All NMR experiments were conducted at 308 K. The 1H,15N-HSQC spectra were acquired with 144 scans, using 200 complex points in the 15N (indirect) dimension and 2048 complex points in the 1H (direct) dimension. Chemical shift perturbations in the backbone amide cross peaks were identified by comparing the 1H,15N-HSQC spectrum recorded for 15N-labeled Gal9 in the apo form with those obtained in the presence of the peptides. Different cross peaks belonging to amino acid residues of both domains were unambiguously identified.

Immunofluorescence and confocal microscopy

Day 6 DCs were treated with 1 µg/mL Staphylococcal enterotoxin B or super antigen B (#S4881, Sigma Aldrich) for 1 h at 37ºC (5% CO2). After extensive washing, 60.000 mature WT or gal-9 depleted DCs were incubated in 96-well low attachment plates with autologous T cells (1:2 ratio) for 2 h prior to being transferred to a 12 mm PLL-coated coverslip (#P4707-50 ml, Sigma-Aldrich). Cells were left to adhere for 10 min prior to being fixed in 4% PFA for 10 min at RT and subsequently incubated for 20 min in 0.1M quenching solution (NH4Cl in PBS). Coverslips were washed twice with PBS, then either permeabilized (2.5% donkey serum, 0.1% saponin) for intracellular staining or left non-permeabilized (2.5% donkey serum) for surface staining. All samples were incubated overnight at 4°C with anti-gal9 (#AF2425, R&D biosystems, 1:40). The following day, coverslips were incubated for 1 h in permeabilization buffer at RT, in the dark with the following antibodies: donkey-anti-goat alexa 568 (#A11055, ThermoFisher Scientific) at 1:400 (v/v) dilution, anti- human TCR alpha (α) and beta (β) FITC (#43720, BD Biosciences) at 1:50 (v/v) or anti-human CD3 BV421 (#344833, Biolegend, clone SK7) at 50µg/mL, anti- human HLA-(DR/DP/DQ) Alexa flour 647 (#563591, BD Biosciences, clone Tü39) at 1:50 (v/v). After incubation, cells were washed thoroughly with PBS, incubated with 0.3µg/ml 4´-6-diamidino-2-phenylindole (DAPI 1:3000 dilution) for nuclear staining, washed again with PBS and embedded in glass slides using 8 µl Mowiol (Calbiochem). Coverslips were allowed to dry overnight at RT in the dark and stored at 4°C until imaging. Samples were imaged with both a Leica DMI6000 epi-fluorescence

microscope fitted with a 63 × 1.4 NA oil immersion objective, a metal halide EL6000 lamp for excitation, a DFC365FX CCD camera and GFP and DsRed filter sets (all from Leica, Wetzlar, Germany) as well as a Zeiss LSM900 confocal laser scanning microscope equipped with the Airyscan module. For the images acquired with the Leica DMI6000 epi-fluorescence microscope we used a 63 × 1.4 NA oil immersion objective, a metal halide EL6000 lamp for excitation, a DFC365FX CCD camera and GFP and DsRed filter sets (all from Leica, Wetzlar, Germany). Focus was kept stable with the adaptive focus control from Leica. For the images acquired with eh Zeiss LSM900 microscope we used a 63 × EC Epiplan-NEOFLOUAR oil immersion objective and three laser module URGB (405, 488, 561, 640 nm). Images were analyzed with Fiji software. Fluorescence enrichment was quantified using a custom-made macro in Fiji ImageJ. The Mander's and Pearson's correlation coefficients were calculated using the JACoP plugin and the line scan graphs representing the fluorescence cross-sections were quantified using the Plots profile command in Fiji ImageJ and depicted using GraphPad Prism 8 or 10 software. The cross-sections were established by drawing a perpendicular line from the center of the immune synapse towards the plasma membrane at the rear of the cell.

MHC-II immunopeptidomics

MHC-II-bound peptides from WT and KD gal9 DCs were eluted as described in the Materials and Methods section. Before mass spectrometry, peptides were eluted from StageTips into protein LoBind tubes using 30 µL of buffer B (80% acetonitrile, 20% LC-grade water, 0.01% formic acid), followed by drying in a vacuum centrifuge at 45 °C. The immunopeptidomes were then resuspended in 7.5 µL buffer A and half of the sample was used for measurement by single-shot LC-MS/MS on the Orbitrap Astral mass spectrometer (Thermo Scientific) connected to a Vanquish Neo nano-LC system (Thermo Scientific) using a 30 samples-per day (30SPD) LC-MS method. The Vanquish Neo was operated in trap-and-elute mode with peptides loaded onto a PepMap Neo C18 5µm trap column (300µm x 5mm, Thermo Scientific) before separation on the AUR3-25075C18-XT (1.7µm/75µm x 25cm, IonOpticks) column. The column was heated at 50°C and mounted onto an Easyspray ion source (Thermo Scientific) with ion spray voltage set to 1500V or higher. Solvent A was 0.1% formic acid/water and solvent B was 0.1% formic acid/80% acetonitrile in water and peptides were separated at flow rates of 0.4 µl/min in a circa 36-min effective gradient containing a non-linear increase from 8% to 45% solvent B, followed by a wash-out at 99% solvent B and equilibration at the end using the "fast equilibration" script in combined control mode with a 1450 bar pressure limit. A DDA method was used with full MS resolution in the Orbitrap set to 240,000 and peptide precursors with charge states 1-6 being sampled for MS/MS in 0.6 sec. cycles. MS1 mass range was 400-1500 m/z, the normalized AGC target was 500% and maximum IT was set to 40 ms. For MS2, the intensity threshold was 5×103 and a 20 sec. exclusion duration was used. Precursors isolated in the quadrupole within a 1.2 m/z isolation window were fragmented with a normalized HCD collision energy of 25% and MS2 spectra were acquired in the Astral analyzer with scan range 120-1800 m/z, 200% normalized AGC target and a 30 ms maximum injection time.

The DDA RAW files were searched using FragPipe (v23.1) using the built-in 'Nonspecific-HLA-C57' workflow with all preset parameters. MS data was searched using MSFragger1 . The initial precursor and fragment mass tolerances were set to 20 ppm. Spectrum deisotoping, mass calibration, and parameter optimization were enabled. The isotope error was set to "0/1". The tetanus toxin (UniProt ID: C4PD05) and ovalbumin (UniProt ID: P01012) protein sequences were spiked into the reviewed Homo sapiens protein sequence database obtained from UniProt (downloaded on 19 August 2025), appended with common contaminants and decoys for use in the search. The enzyme cleavage was set to "nonspecific" and the peptide length was restricted to 7–25. Carbamidomethylation of cysteine was specified as a fixed modification. Cysteinylation, oxidation of methionine, N-terminal acetylation, and Pyro-glu from E and Q were set as variable modifications. MSBooster and Percolator were used to predict the RT and MS/MS spectra, and to rescore PSMs2 , followed FDR filtering by Philosopher3 . A protein FDR filter was not applied. The 'combined_peptide.tsv' text file was used for downstream analysis and plotting in R (v4.3.1).

Figures

Fig. S1.

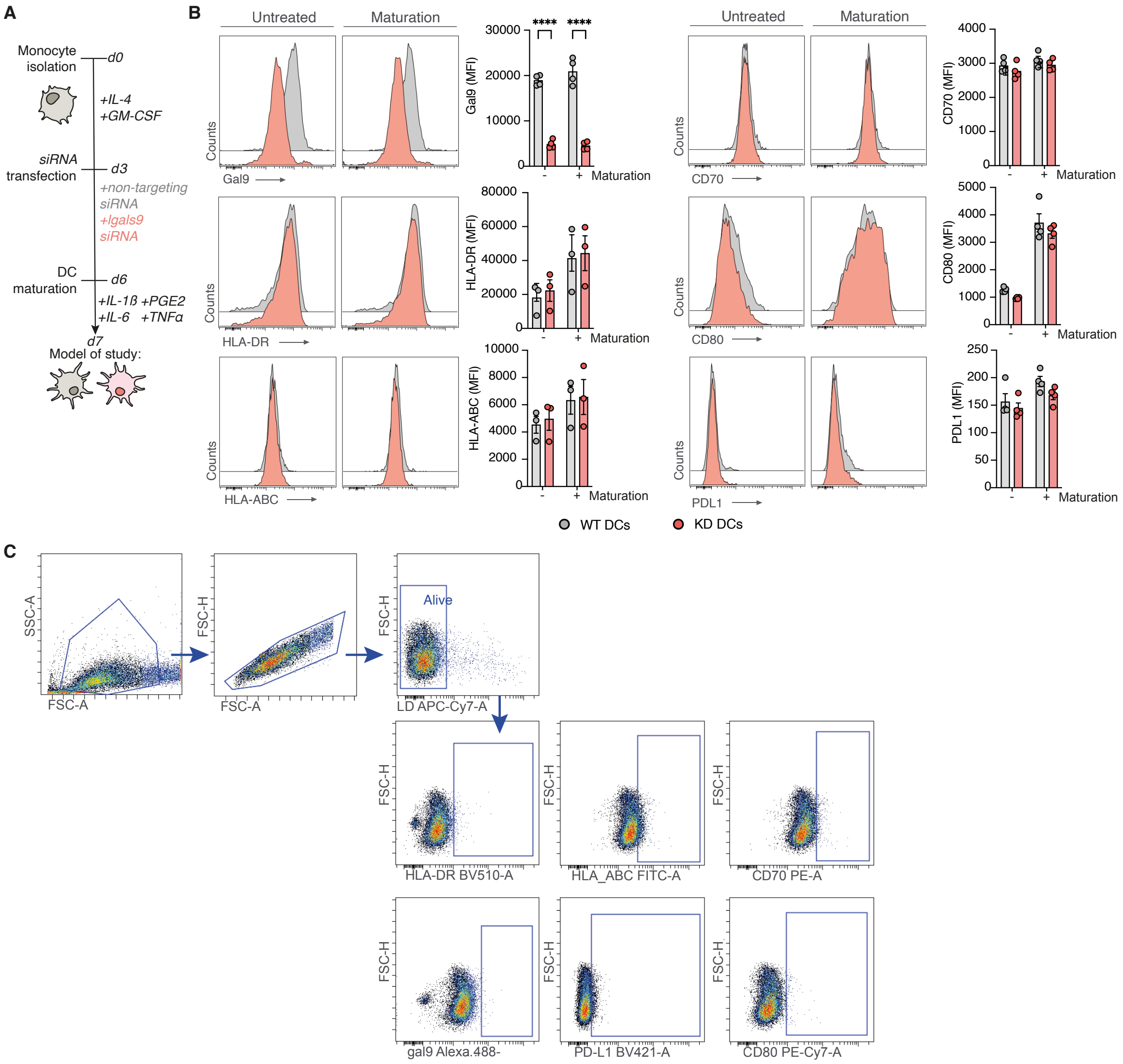

Human DCs depleted for gal9 are not impaired in their maturation. A. Graphical representation of the experimental timeline for WT and gal9 KD DC generation. DCs were generated by isolating CD14 $^{+}$ cells from total PBMCs and treated with IL-4 and GM-CSF for three days prior to being transfected with a non-targeting or a lgals9 siRNA (hereby referred as wildtype, WT, or knockdown, KD gal9, respectively). After day 6 of culture, DCs were matured with a mixture of pro-inflammatory cytokines (IL-6, IL-1β, TNFα and PGE2) for 24 h. These cells were used as a model system to study the role of gal9 in human DCs. **B.** WT (grey) or KD gal9 (red) DCs were matured with IL-1β, IL-6, PGE2, and TNFα during 18–24 h, and membrane expression of gal9, HLA-DR, CD70, CD80, HLA-ABC, and PDL1 was assessed by flow cytometry. Flow cytometry histograms are shown. Graph represent the mean fluorescence intensity (MFI) ± SEM from four donors. Two-way ANOVA with Šídák's multiple comparisons test was conducted between conditions. ****p < 0.0001. **C.** Gating strategy employed to analyze data in for (B). Dot plots represent the fluorescence minus one (FMO) staining controls.

Fig. S2.

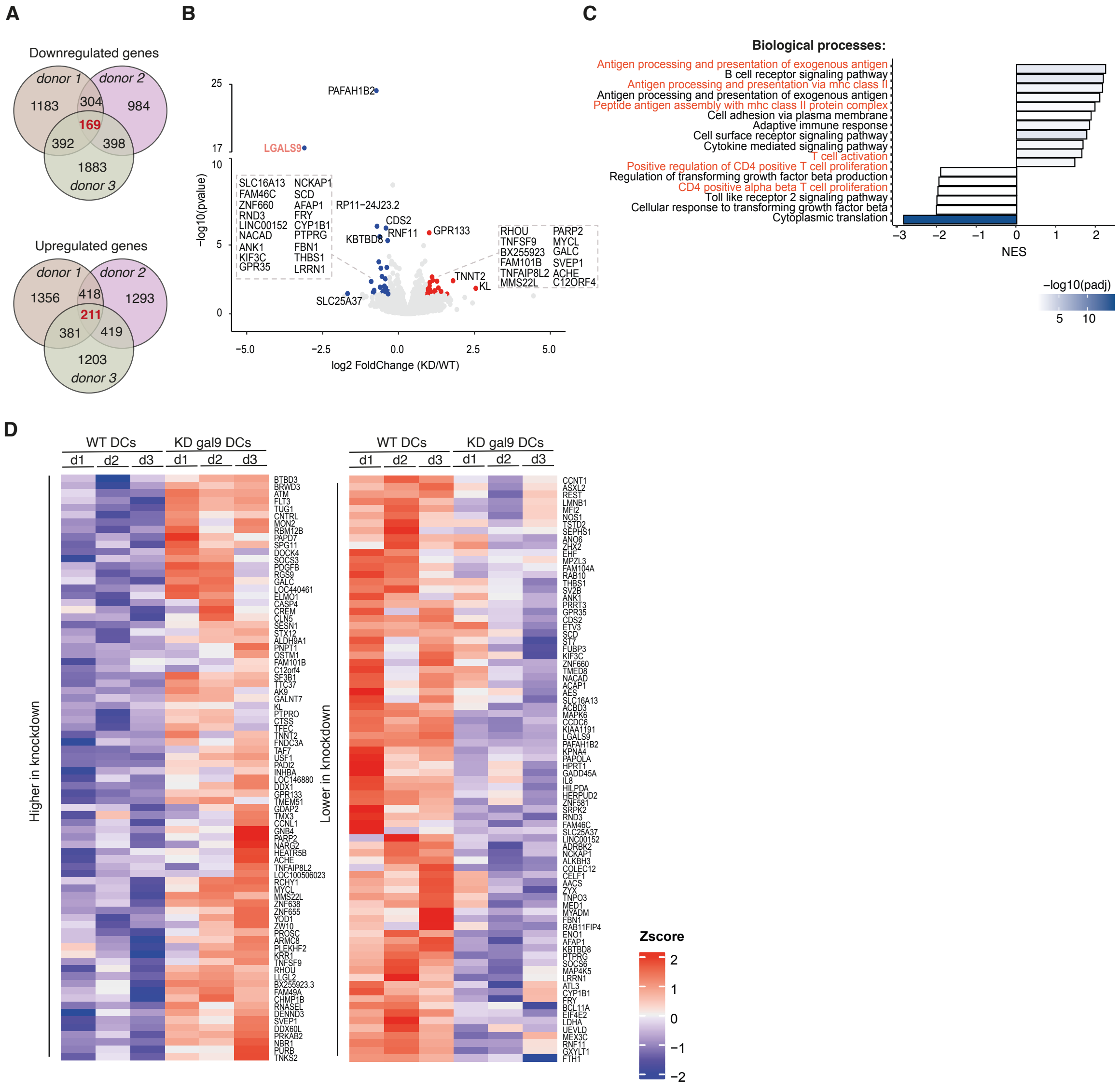

Transcriptionally, gal9-depleted DCs display altered T cell proliferation and MHC-II complex assembly pathways. A. Venn diagrams showing overlap in gene expression changes among the three donors analyzed. The top diagram represents the number of genes commonly upregulated across all donors, while the bottom diagram displays downregulated genes. The number in red indicates the genes commonly altered across all three donors and used for analysis. **B.** Volcano plot of differentially expressed genes comparing KD gal9 to WT conditions. Red dots denote significantly upregulated genes, and blue dots significantly downregulated genes.

Differentially expressed gene names are indicated next to the dot or in a squared gray dotted box (lgals9 gene is marked in red). Fold change represents KD gal9 vs. WT DC expression levels. **C.** Gene set enrichment analysis using gene ontology (GO) terms from the Biological Process dataset performed on gProfiler. Color intensity corresponds to the -log adjusted p-value, with darker colors indicating greater significance in pathway enrichment. MHC-II and T cell activation and proliferation-related pathways are highlighted in red. **D.** Heatmap showing Z-transformed gene expression values for all genes altered in the gal9 KD cells compared to WT controls.

Fig. S3.

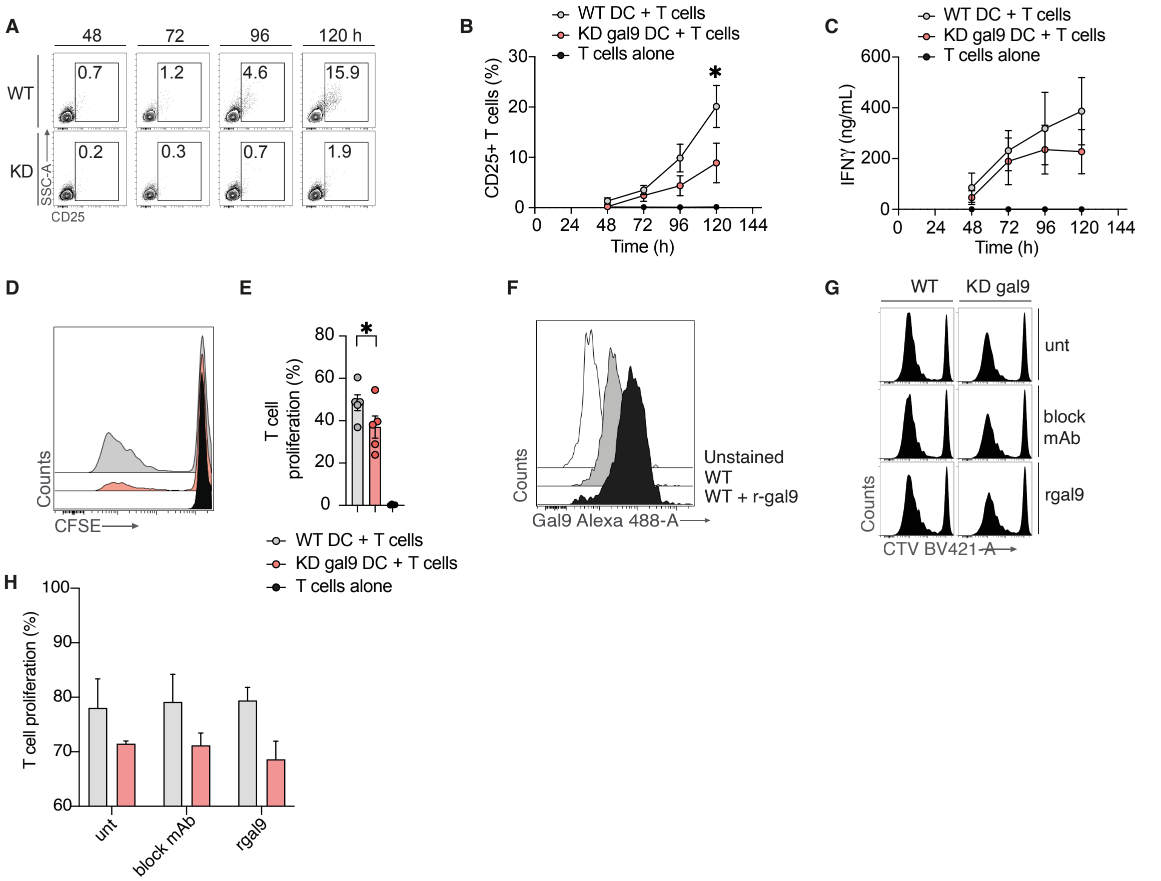

Intracellular gal9 regulates DC-mediated allogeneic T cell effector functions. A. Representative flow cytometry plots depicting CD25 expression on T cells cultured alone (black) with WT (grey) or KD gal9 (red) DCs over 48-120h. Squares and number in inset refer to % of CD25+ cells. **B.** Quantification of (A) from four independent donors. **C.** IFN-γ (ng/mL) secretion by T cells under the same conditions. **D.** Representative flow cytometry histogram of allogeneic CFSE-labeled T cells cultured as in (B). **E.** Percentage of proliferating T cells (D) from 5 donors. **F.** Flow cytometry histograms depicting gal9 mean fluorescence intensity in unstained (white), WT (grey), and recombinant gal9-treated WT (black) DCs. **G.** Representative proliferation histogram of T cells cultured with WT or KD gal9 DCs untreated or treated with either anti-gal9 blocking antibody or with recombinant gal9 for 1 hour. **H.** Percentage of allogeneic CTV-labeled proliferating T cells of data shown in (G) from two independent donors. Grey = T cells co-cultured with WT DCs. Red = T cells co-cultured with KD gal9 DCs. Data are mean ± SEM (n=2–5). Statistical significance assessed by two-way ANOVA with Šídák's multiple comparisons (B, C, H) or paired t-test (E). *$p < 0.05$.

Fig. S4.

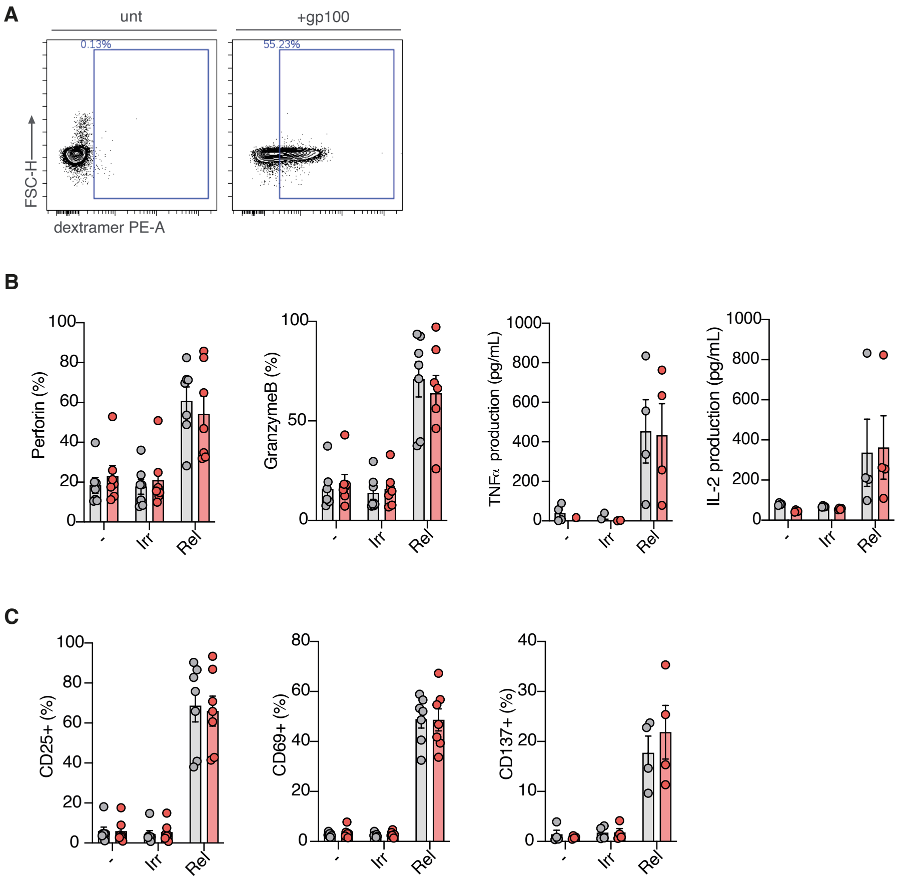

Gal9-deficient DCs do not alter CD8+ T cell activation and cytotoxic capacity. A. Dot plots depicting gp100 dextramer staining in CD8+ T cells after 5 h of being of transfected with a gp100-specific TCR-encoding mRNA. **B.** Quantification of perforin, granzyme B, TNFα, and IL-2 production, from left to right (as % positive CD8+ T cells) for 4–7 independent donors. **C.** Quantification of CD25, CD69 and CD137 expression, from left to right (as % positive CD8+ T cells) for 4–7 independent donors. All bar graphs show mean ± SEM. Each dot represents one independent donor; T cells cultured with WT DCs (grey) or KD gal9 DCs (red). Two-way ANOVA with Šídák's multiple comparisons test was performed between conditions.

Fig. S5.

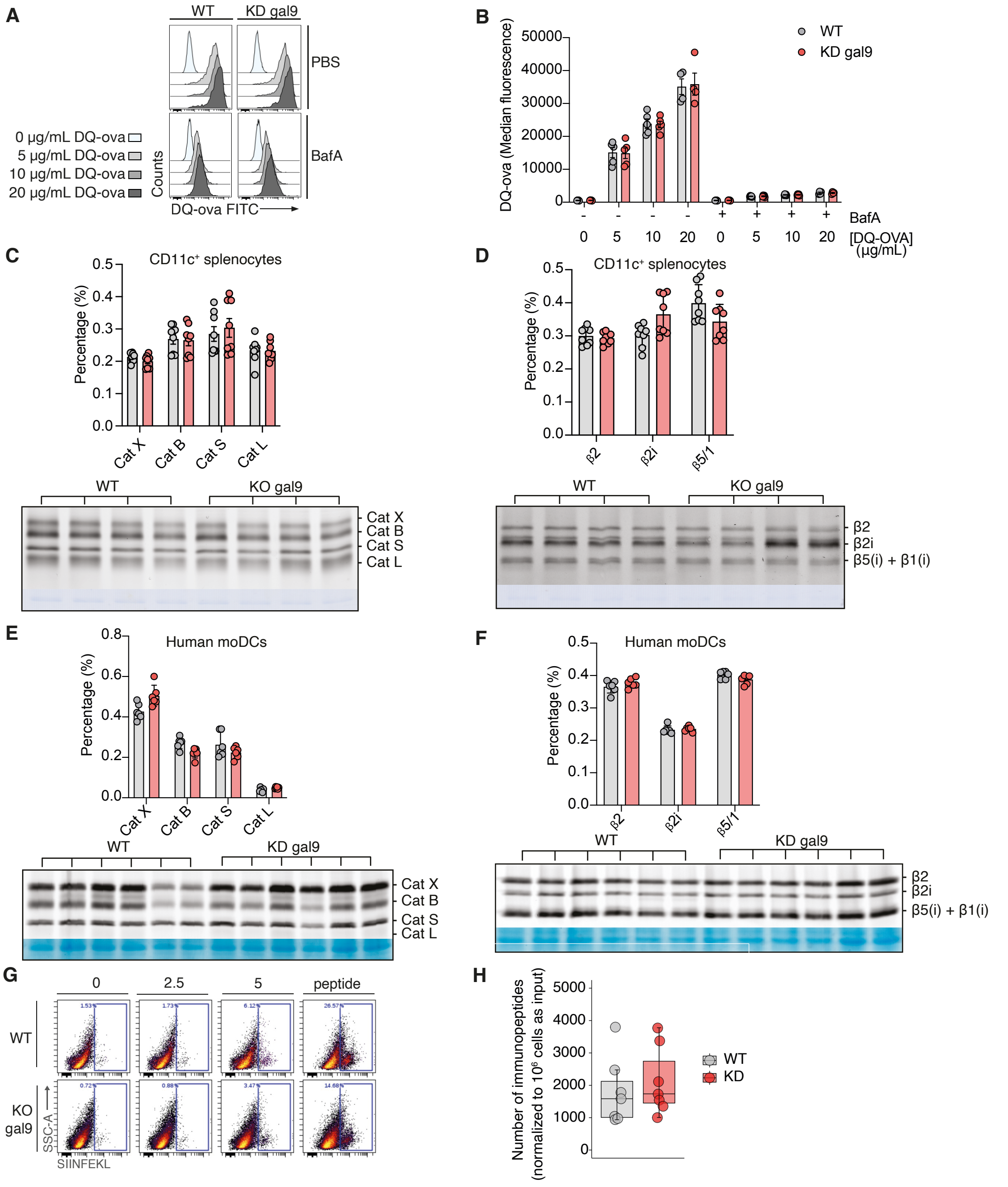

Gal9-deficient DCs do not display differences in OVA processing, presentation or cathepsin and proteasome subunit expression. A. Flow cytometry histograms depicting the fluorescence intensity of the DQ-OVA probe (0, 5, 10, 20 µ g/mL) in WT or KD gal9 DCs treated with PBS or Bafilomycin A (BafA) as inhibitor for lysosome acidification. **B.** Quantification of data shown in (A) from five independent donors. Grey: WT DCs. Red: KD gal9 DCs. **C.** Cathepsin profiling of WT and gal9 KO CD11c^{+} splenocytes. Representative SDS-PAGE is shown with cathepsins assigned. Quantification done for four mice (technical duplicates). **D.** Proteasome profiling of WT and gal9 KO CD11c^{+V} splenocytes. Representative SDS-PAGE is shown with proteasome subunits assigned. Quantification done for four mice (technical duplicates). **E.** Cathepsin profiling of WT or KD gal9 DCs. SDS-PAGE is shown with cathepsins assigned.Quantification done for three independent donors (technical duplicates). **F.** Proteasome

profiling of WT or KD gal9 DCs. SDS-PAGE is shown with proteasome subunits assigned. Quantification done for three independent donors (technical duplicates). **G.** Dot plot showing fluorescence intensity of the SIINFEKL peptide bound to MHC-I detected in WT and gal9 KO murine DCs after culturing them with 0, 2.5 or 5 mg/mL OVA (or peptide as positive control) at 37°C for 18 h. **H.** Number of peptides identified in the WT and KD gal9 DCs, normalized to the number of cells used as input. WT (grey) or KO/KD gal9 (red) DCs. Data shown as mean ± SEM (in B and H) or SD (in C, D, E and F). Two-way ANOVA with Šídák's multiple comparisons test was conducted between conditions.

Fig. S6.

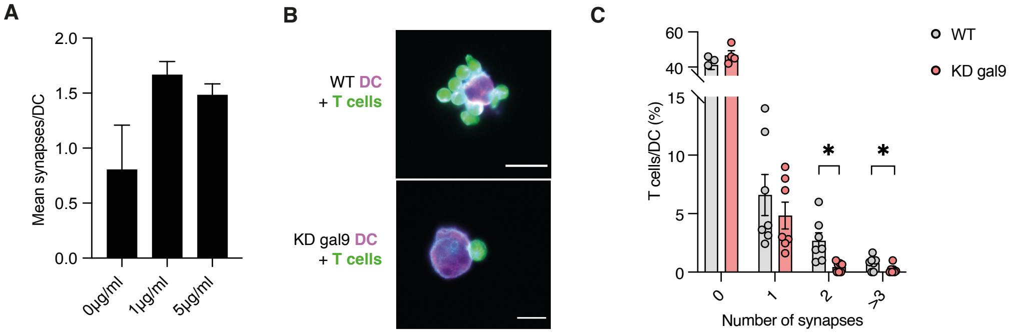

Immune synapse formation is impaired in gal9-deficient DCs A. Histogram showing the amount of DC-T cells synapses/DC tracked using live cell microscopy after 75 minutes. DCs were treated with 0, 1 or 5 µg/mL of superantigen (synapse inducer). **B.** Allogeneic WT and KD gal9 DCs (magenta) were co-cultured with T cells (green) for 2 hours in low-adhesion plates. Following incubation, cells were fixed and transferred to an imaging plate for visualization. Scale bars = 10 µm. **C.** Quantification of the number of WT or KD gal9 DCs establishing contact with allogeneic T cells (0, 1, 2 or >3 T cells/DC) of data shown in (B) for 4–7 independent donors (>50 DCs -with or without synapses- were quantified per donor). Grey = T cells co-cultured with WT DCs. Red = T cells co-cultured with KD gal9 DCs. Two-way ANOVA with Šídák's multiple comparisons test was conducted between conditions. *$p < 0.05$. Graphs depict mean values ± SEM.

Fig. S7.

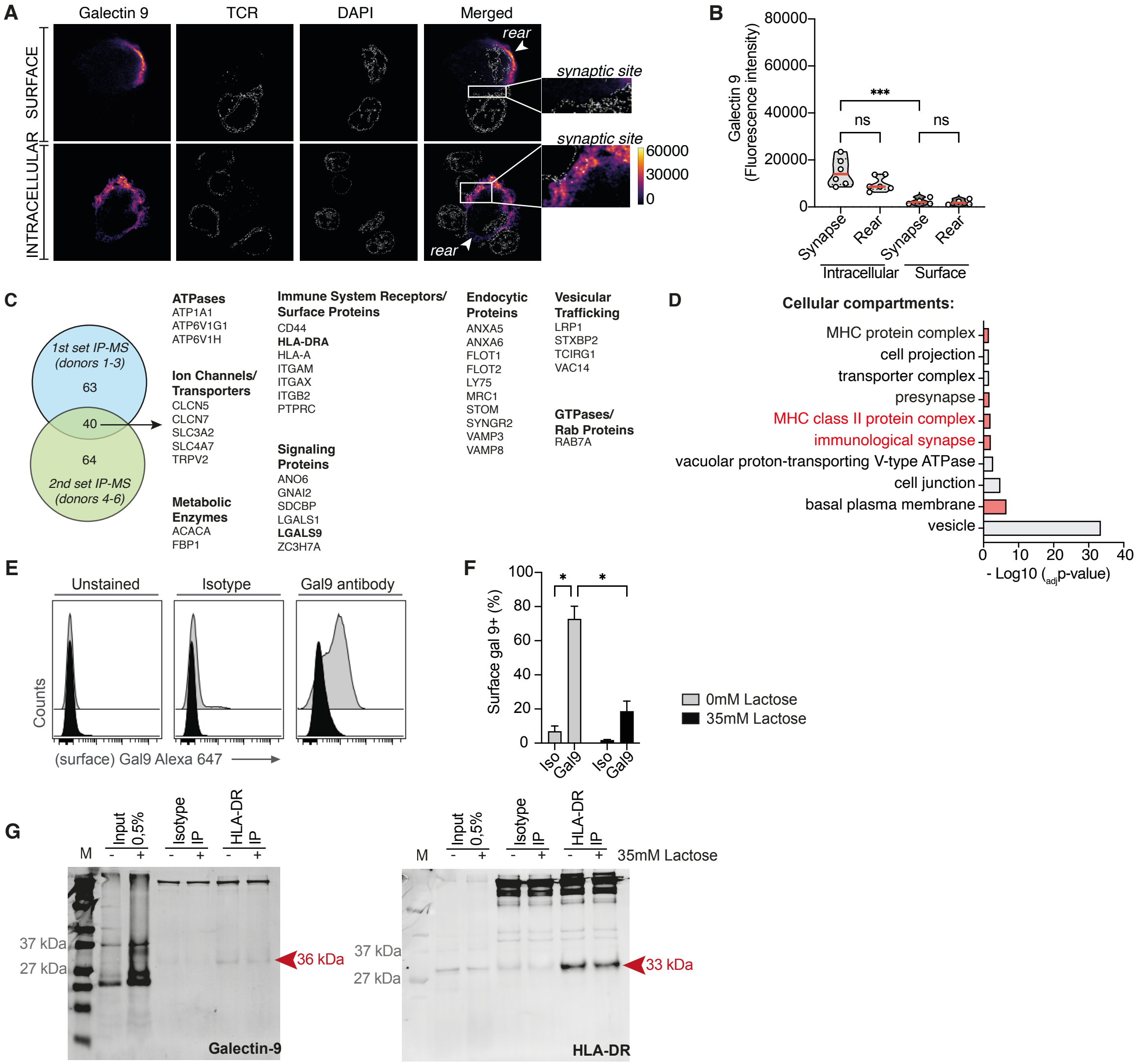

Gal9 co-immunoprecipitated proteins associate with MHC-II complex assembly and immune synapse pathways. A. Representative microscopy images of WT DCs incubated for 2 h with T cells before being fixed, permeabilized (intracellular, bottom) or not (surface, top) and stained for gal9, CD3 and DAPI. A merged composite image is also shown. Fluorescence intensity is depicted as colorized pixels, with grayscale values representing signal intensity. The zoomed-in image (2×) highlights the localization of gal9 at the DC–T cell interface and the white arrows indicate the rear of the cell. Scale bars = 10 µm. **B.** Quantification of fluorescence intensity values of gal9 at the immune synapse compared with the rear of WT DCs co-cultured with T cells. Graph depicts mean ± SEM for 4-6 independent donors. Ordinary one-way ANOVA analysis with Tukey's multiple comparisons test was performed; ns $p > 0.05$, ***$p < 0.001$. **C.** Venn diagram showing the overlap of gal9 binding proteins identified from two sets of mass spectrometry–immunoprecipitation experiments, each performed with three independent donors. A total of 40 common hits independently enriched at least 1.5-fold between gal9 and isotype pull-downs are shared between the two experiments. The list of gal9 binding proteins (gene names) is categorized by their respective biological functions. **D.** Cellular compartments related-pathways significantly enriched related to gal9-interacting proteins identified in C. Pathways related to MHC-II complex and immune synapse are highlighted in red. Statistical significance is represented by the adjusted p-value (−log10) (x-axis). **E.** Representative histograms of lactose

(un)treated (0 mM in grey and 35 mM in black) DCs. Surface galectin-9 depletion was measured after 30 minutes of treatment by flow cytometry. Isotype control was taken along. **F.** Quantification of (E) as the percentage of galectin-9+ DCs after lactose treatment (three independent donors). Data represents mean ± SEM. *p < 0.05. **G.** Representative western blot (WB) of galectin-9 and HLA-DR following immunoprecipitation of HLA-DR from DC lysates (0 or 35mM lactose treated). Immunoprecipitation was performed using an anti-HLA-DR antibody or IgG as negative control; and immunoprecipitated complexes were resolved by WB and probed with antibodies against HLA-DR and galectin-9. Input (0,5% of total lysate) and IgG isotype controls are shown for comparison. M = protein ladder. Red arrow indicates the band of interest.

Fig. S8.

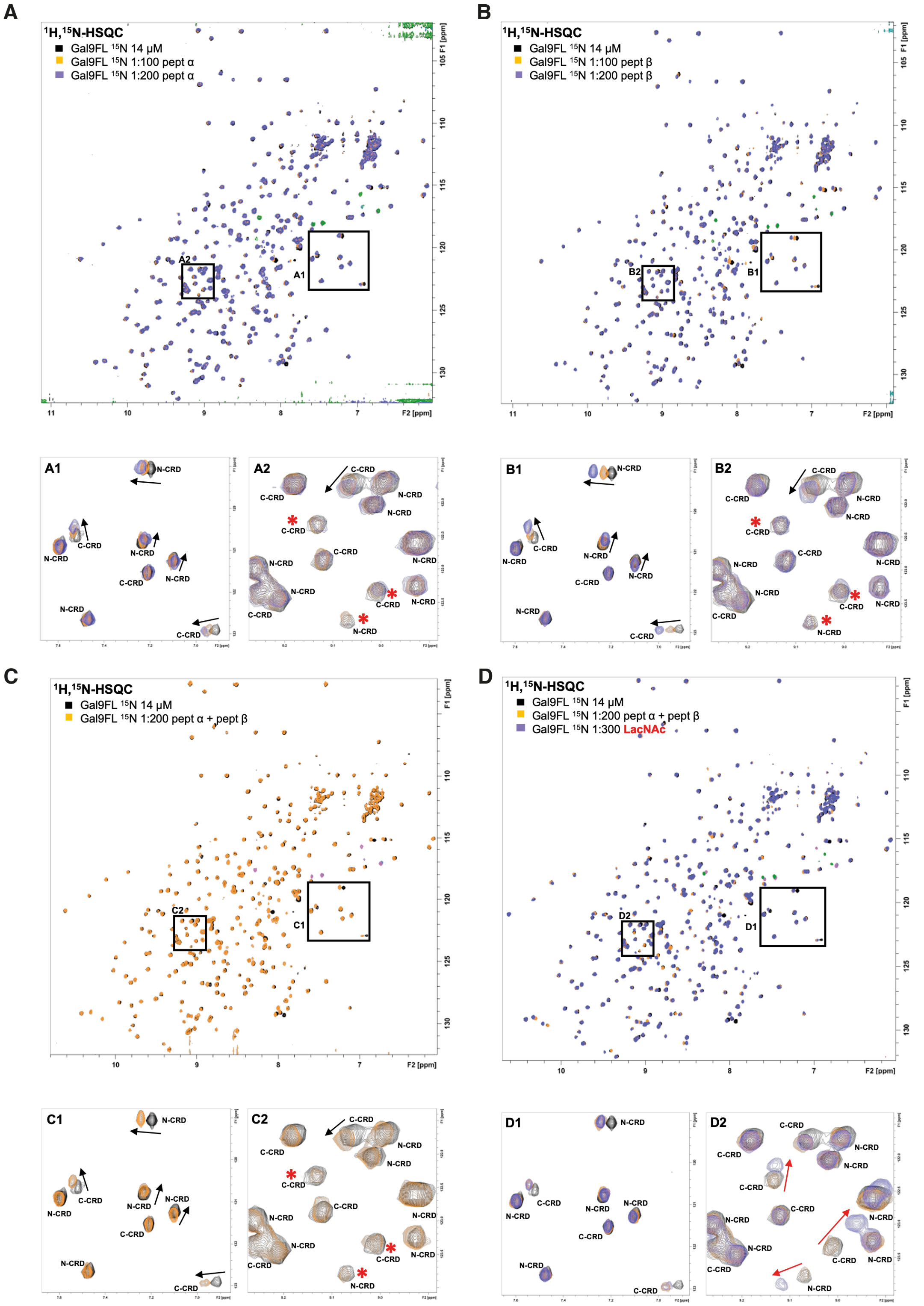

Gal9 binds intracellular α and β HLA-DR domains independently of glycan-mediated lactose interaction. ^{15}N-gal9 (14uM) was titrated with: **(A)** 100 and 200 molar equivalents of cytosolic HLA-DR-α peptide **(B)** 100 and 200 molar equivalents of cytosolic HLA-DR-β peptide

and (**C**) 200 molar equivalents of a mixture of cytosolic HLA-DR-α + HLA-DR-β peptides. (**D**) 300 molar equivalents of LacNAc were added to the gal9 + peptides sample in (C). Two regions are zoomed-in below each $^{1}H,^{15}N$-HSQC spectra. Black arrows indicate the chemical shift perturbation of crosspeaks due to the interaction with the peptides. Red arrows arrows indicate the chemical shift perturbation of crosspeaks due to the interaction with LacNAc. Red Asterisks indicate crosspeaks that are perturbed due to the interaction with LacNAc but not with the peptides.

Fig. S9.

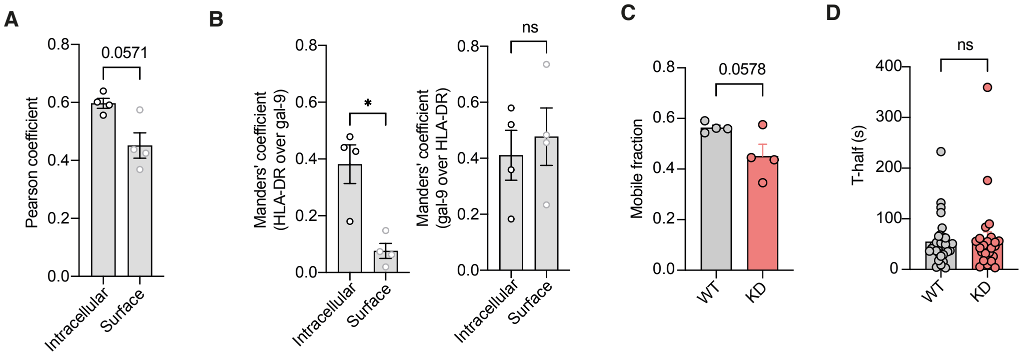

Gal9 colocalizes intracellularly with HLA-DR and regulates its recruitment to the synapse and membrane lateral mobility. A and **B.** Pearson's (A) and Manders' (B) correlation coefficient for HLA-DR and gal9 from images shown in (Figure 6 A). Graph depicts mean values ± SEM for 4 independent donors. Intracellular (permeabilized) and surface (non-permeabilized) stainings were compared using a Mann-Whitney statistical test. ns $p > 0.05$, *$p < 0.05$. **C.** Quantification of the mobile fractions of HLA-DR in WT (grey) and gal9 KD DCs (red). Each dot represents the average values of all cells form the individual donor (n=4). **D.** Recovery kinetics of HLA-DR in DCs, shown as half-time (T½) in seconds. Each dot represents a different cell, ns $p > 0.05$. FRAP experiments (C and D) were performed 4 independent donors (6-15 cells/donor) and statistical significance was assessed using a Mann-Whitney test, mean ± SEM is shown. ns $p > 0.05$.

Fig. S10.

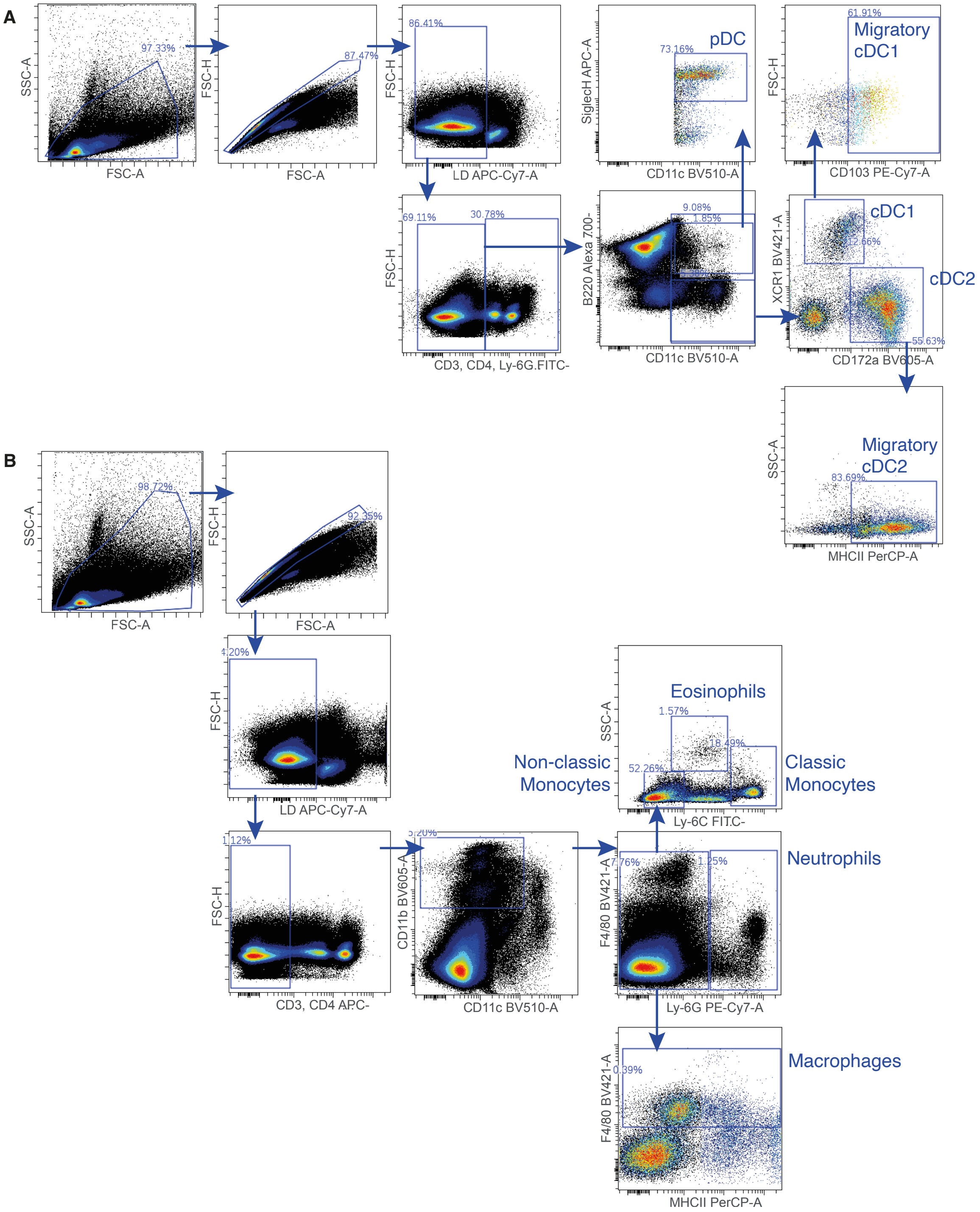

Flow cytometry gating strategy for DC subsets and myeloid populations in naïve-treated animals.

Fig. S11.

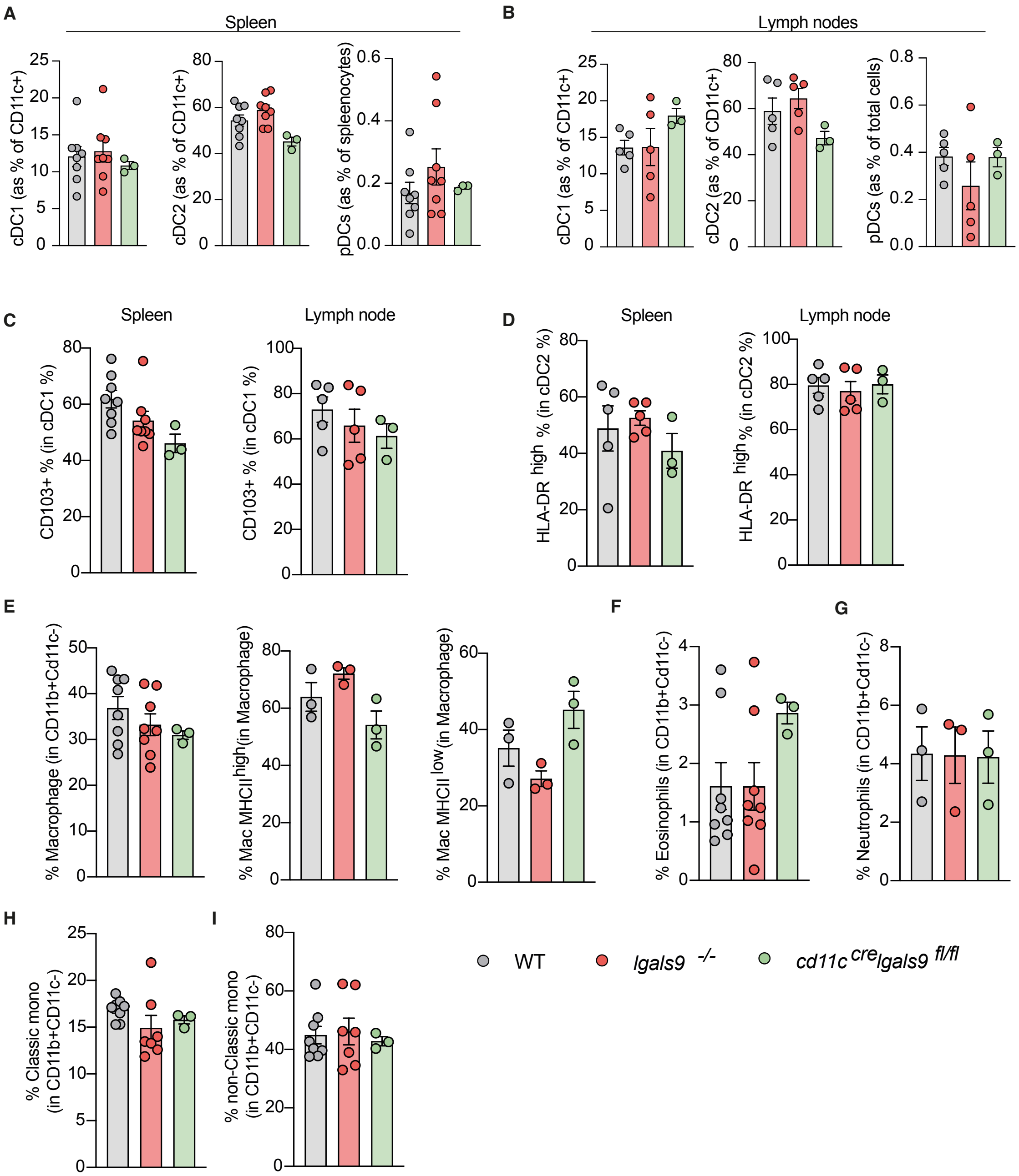

Gal9 does not regulate dendritic cell development. A and B. Percentage (%) of DC subsets in the spleen (A) and lymph nodes (B) of WT (grey), total gal9 KO (red), and conditional gal9 KO in DCs (green). cDC1s and cDC2s are defined as XCR1+CD172- and XCR1-CD172+ cells, respectively, within the CD11c+B220- population. pDCs are defined as SiglecH+ CD11c+B220+ cells, within the alive population (spleenocytes for spleen samples or total cells for lymph node samples). **C–I.** Percentage (%) of DC subsets and myeloid cell types in the spleen and lymph nodes of three mouse models. Data represent mean ± SEM of 3–8 independent animals. One-way ANOVA with Dunnett's multiple comparisons test was conducted to compare conditions.

Fig. S12.

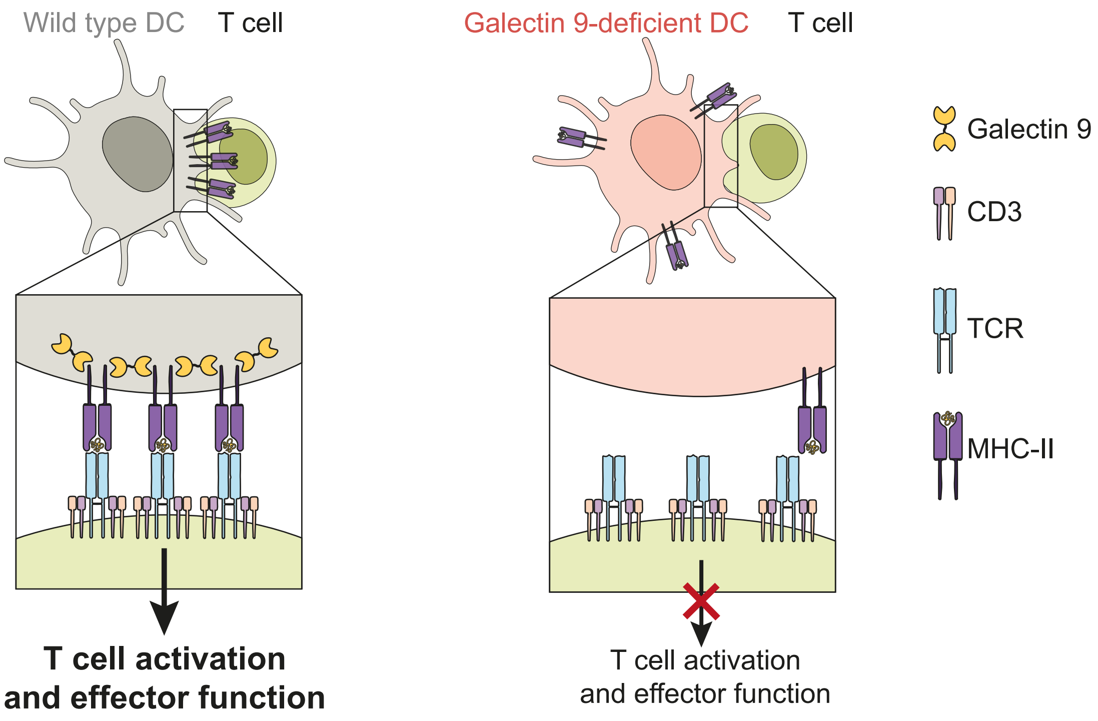

Graphical abstract. Gal9 enables immune synapse formation between DCs and T cells. Intracellular gal9 binding to HLA-DR drives its recruitment to the immune synapse, enabling efficient DC–T cell engagement resulting in T cell activation and effector function (left). In the absence of gal9, HLA-DR fails to be recruited to the immunological synapse, impairing DC-mediated T cell activation (right).

Tables

Table S1. Antibody list

Application	Antibody Target	Company	Catalog Number	Clone	Color	Dilution
Human DC maturation	HLA-DR	BD BioSciences	# 555811	G46-6	FITC	1:50
	HLA-DR	BioLegend	# 307646	L243	BV-510	1:50
	CD40	BD BioSciences	# 561215	5C3	PE-Cy7	1:50
	CD80	BD BioSciences	# 557227	L307.4	PE	1:50
	CD80	BD BioSciences	# 561135	L307.4	PE-Cy7	1:50
	CD86	BD BioSciences	# 555660	2331	APC	1:50
	CD11b	BioLegend	# 301342	ICRF44	APC-Cy7	1:50
	HLA-ABC	BD BioSciences	# 555553	G46-2.6	PE	1:50
	HLA-ABC	Miltenyi	# 130-101-466	REA230	APC	1:50
	PDL-1	BD BioSciences	# 563738	MIH1	BV-421	1:50
	CD70	BD BioSciences	# 555835	Ki-24	PE	1:50
	CD14	BD BioSciences	# 560180	MoP9	APC-H7	1:50
Human T cell proliferation	CD25	BD BioSciences	# 555434	M-A251	APC	1:25
	CD69	BD BioSciences	# 555530	FN50	FITC	1:25
	CD8	BioLegend	# 344708	SK1	PerCP	1:25
	CD3	eBioscience	# 25-0038-42	UCHT1	PE-Cy7	1:25
	CD4	BD BioSciences	# 560158	RPA-T4	APC-H7	1:25
Mouse dendritic cell panel	XCR1	Biolegend	# 148216	ZET	BV421	1:50
	CD11c	Biolegend	# 117353	N418	BV510	1:50
	CD172a	Invitrogen	# 17-1721-82	P84	BV605	1:50
	CD3	Biolegend	# 100204	17A2	FITC	1:50
	CD4	Biolegend	# 100510	RM4-5	FITC	1:50
	Ly6G	Biolegend	# 127606	1A8	FITC	1:50
	Gal9	Biolegend	# 136101	RG9-35	PE	1:25
	MHCII	Biolegend	# 107624	M5/114.15.2	PerCP	1:50
	CD103	Biolegend	# 121426	2E7	PeCy7	1:50
	SiglecH	eBioscience	# 51-0333-82	eBio440c	af647	1:50
Mouse myeloid panel	F4/80	Biolegend	# 123131	BM8	BV421	1:50
	CD11c	Biolegend	# 117353	N418	BV510	1:50
	CD11b	BD	# 553309	M1/70	BV605	1:50
	Ly-6C	BD	# 553104	AL-21	FITC	1:50
	Gal9	Biolegend	# 136101	RG9-35	PE	1:25
	MHCII	Biolegend	# 107624	M5/114.15.2	PerCP	1:50

	Ly-6G	Biolegend	# 127618	1A8	PeCy7	1:50
	CD3	Biolegend	# 100312	145-2C11	APC	1:50
	CD4	Biolegend	# 100516	RM4-5	APC	1:50

Movie S1 and S2 (separate file). Live cell imaging movies of dendritic cell (DC) and T cell co-cultures. Movie 1. Wild-type (WT) DCs (magenta) interacting with T cells (green). **Movie 2.** Gal9 KD DCs (magenta) interacting with T cells (green). Time progression is indicated by the yellow timestamp (in minutes).

SI References

1. MSFragger: ultrafast and comprehensive peptide identification in mass spectrometry-based proteomics - PubMed. https://pubmed.ncbi.nlm.nih.gov/28394336/.
2. Yang, K. L. et al. MSBooster: improving peptide identification rates using deep learning-based features. Nat Commun 14, 4539 (2023).
3. da Veiga Leprevost, F. et al. Philosopher: a versatile toolkit for shotgun proteomics data analysis. Nat Methods 17, 869–870 (2020).
